# The developmental and genetic bases of apetaly in *Bocconia frutescens* (Chelidonieae: Papaveraceae)

**DOI:** 10.1186/s13227-016-0054-6

**Published:** 2016-08-02

**Authors:** Cristina Arango-Ocampo, Favio González, Juan Fernando Alzate, Natalia Pabón-Mora

**Affiliations:** 1 Instituto de Biología, Universidad de Antioquia, Medellín, Colombia; 2 Instituto de Ciencias Naturales, Universidad Nacional de Colombia, Bogotá, Colombia; 3 Centro de Secuenciación Genómica Nacional (CSGN), Sede de Investigación Universitaria, Universidad de Antioquia, Medellín, Colombia

**Keywords:** ABCE model, *AGAMOUS*, *APETALA3*, Apetaly, *Bocconia*, Homeosis, *Macleaya*, Papaveraceae, *Stylophorum*

## Abstract

**Background:**

*Bocconia* and *Macleaya* are the only genera of the poppy family (Papaveraceae) lacking petals; however, the developmental and genetic processes underlying such evolutionary shift have not yet been studied.

**Results:**

We studied floral development in two species of petal-less poppies *Bocconiafrutescens* and *Macleayacordata* as well as in the closely related petal-bearing *Stylophorum diphyllum*. We generated a floral transcriptome of *B. frutescens* to identify MADS-box ABCE floral organ identity genes expressed during early floral development. We performed phylogenetic analyses of these genes across Ranunculales as well as RT-PCR and qRT-PCR to assess loci-specific expression patterns. We found that petal-to-stamen homeosis in petal-less poppies occurs through distinct developmental pathways. Transcriptomic analyses of *B. frutescens* floral buds showed that homologs of all MADS-box genes are expressed except for the *APETALA3*-*3* ortholog. Species-specific duplications of other ABCE genes in *B. frutescens* have resulted in functional copies with expanded expression patterns than those predicted by the model.

**Conclusions:**

Petal loss in *B. frutescens* is likely associated with the lack of expression of *AP3*-*3* and an expanded expression of *AGAMOUS*. The genetic basis of petal identity is conserved in Ranunculaceae and Papaveraceae although they have different number of *AP3* paralogs and exhibit dissimilar floral groundplans.

**Electronic supplementary material:**

The online version of this article (doi:10.1186/s13227-016-0054-6) contains supplementary material, which is available to authorized users.

## Background

Flowers are evolutionary novelties of nearly 300,000 species. A typical flower has one or two whorls of sterile organs, collectively known as the perianth [1–3], which enclose the stamens and the carpels. Frequently, the perianth consists of an outer series of sepals (collectively termed calyx) and an inner series of petals (collectively termed corolla). Sepals often resemble leaves or bracts in that they are persistent, protect the rest of the floral organs in the bud and often are photosynthetic, while petals are colorful, attract pollinators, are rich in pigments, oils and waxes [3, 4]. The number and morphology of the perianth parts are extremely variable and are often associated with changes in the mechanisms of pollination [5].

**Fig. 1:**
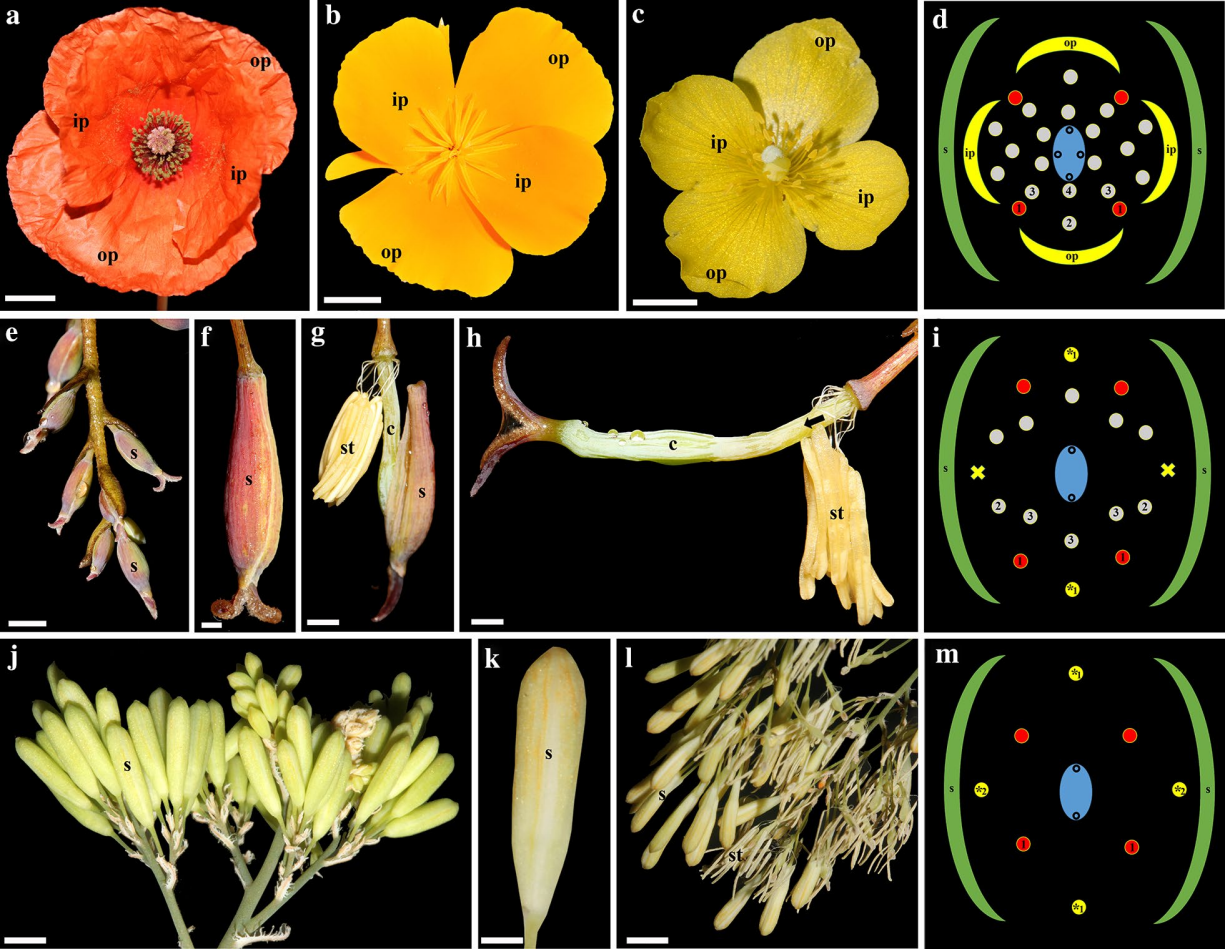
Floral diversity in Papaveraceae. Petal-bearing flowers in *Papaver* *rhoeas* (**a**), *Eschscholzia californica* (**b**) and *Stylophorum diphyllum* (**c**). **d** *S. diphyllum* floral diagram. **e**–**m** Petal-less flowers. **e**–**h** *Bocconia frutescens,* preanthetic and anthetic flowers. **i** *B. frutescens* floral diagram. **j**–**l** *Macleaya cordata*, preanthetic and anthetic flowers. **m** *M. cordata* floral diagram. *Arrow* gynophore; *c* carpel, *ip* inner petal, *op* outer petal, *s* sepal, *st* stamen, * homeotic stamen; 1, first set of 4 true stamens on the first whorl in *red* indicate that these are the first stamens formed and that they occupy the same positions in both petalous and apetalous species serving as reference for the positioning of all other stamens; 2, second set of true stamens of the first whorl; 3, true stamens of the second whorl. 4. True stamens of the third whorl. *Bars* **a**–**c** = 7 mm; **e**, **g**, **h** = 10 mm; **f** = 2 mm, **j**, **l** = 8 mm; **k** = 4 mm

The Ranunculales, the largest order in basal eudicots, exhibit an incredible array of floral forms which range from perianth-less flowers (like in Eupteleaceae) to flowers with bipartite perianths (like in Papaveraceae and Ranunculaceae), sepaloid tepals (like in Menispermaceae) and petaloid tepals (like in Lardizabalaceae and Berberidaceae) [6–8]. With 760 species in 41 genera, the poppy family (Papaveraceae) is the second largest of the order Ranunculales. As currently circumscribed the Papaveraceae s. str. comprise the subfamilies Fumarioideae, with the tribes Fumarieae and Hypecoeae, and Papaveroideae, with the tribes Chelidonieae, Eschscholzieae, Fumarioideae, Papavereae and Platystemoneae [9]. All Papaveraceae exhibit a dimerous floral plan with 2 deciduous sepals, 4 petals in two whorls, many stamens (up to 700) and 2(−8) fused carpels (Fig. 1a–d) [6]. The only exceptions to this floral groundplan are the naturally occurring mutants in *Sanguinaria canadensis* L., which possess eight petals in four whorls as a result of a stamen-to-petal homeosis, and all species in the sister genera *Bocconia* Plum. ex L., and *Macleaya* R. Br., which lack petals likely as a result of a petal-to-stamen homeosis (Fig. 1e–m) [10, 11]. This variation provides an excellent comparative morphological platform for studying the genetic basis of petal identity in non-model organisms. The framework for comparison is the ABCE genetic model of flower development established in *Arabidopsis thaliana* that describes how specific combinations of four classes of MADS-box transcription factors A, B, C and E determine the identity of all floral organs [12–14]. However, all the MADS-box gene lineages have suffered duplications during the course of angiosperm evolution, making the model difficult to extrapolate to non-model flowering plants [15–20]. According to the model, petal identity depends on the combination of A and B and E class genes and the repression of C class genes [12, 14, 21]. Both A and B gene lineages have undergone duplications in the core eudicots, basal eudicots and monocots [16, 22, 23]. In basal eudicots, local duplications in both gene lineages have also been detected [23–25]. A class genes duplicated once, before the diversification of the Ranunculales, resulting in two *FUL*-*like* clades named *RanFL1* and *RanFL2* [24]. Gene copies have been functionally characterized in *Eschscholzia californica* Cham. and *Papaver somniferum* L. (Papaveraceae) and in *Aquilegia coerulea* E. James. (Ranunculaceae). *FUL*-*like* genes in Papaveraceae control flowering time, inflorescence architecture, floral meristem, sepal identity and fruit development. Only *papsfl1-/fl2-*downregulated individuals show atypical green areas in the outer petals, but have normal inner petals [26]. In Ranunculaceae, *AqcFL1A* and *AqcFL1B* (*RanFL1* clade) play roles in inflorescence architecture and leaf morphogenesis, but their role in sepal and petal development is unclear [27]. On the other hand, *AP2*-*like* genes are expressed during carpel, fruit and leaf development and possibly do not control perianth development [23].

B class genes duplicated two times consecutively in the Ranunculales resulting in the *APETALA3I (AP3I)*, *AP3II* and *AP3III* clades [7, 21, 25]. However, the Papaveraceae only have members of the *AP3I* and *AP3III* clades, whereas the Ranunculaceae have gene representatives in all clades [25]. Gene copies have been functionally characterized in *Papaver somniferum,Nigella damascena* and *Aquilegia coerulea* and in general have shown subfunctionalization [25, 28–30]. In *P. somniferumPapsAP3*-*1* is responsible for the identity of petals, whereas *PapsAP3*-*2* controls the identity of stamens. In *A. coerulea*, *AqAP3*-*1* specifies the identity of staminodia, whereas *AqAP3*-*2* is responsible for identity of stamens and *AqAP3*-*3* specifically controls petal identity [25, 29, 31]. Finally, in *N. damascenaNdAP3*-*3* is responsible for petal and outer stamen identity, while *NdAP3*-*1* and *NdAP3*-*2* control stamen identity [30, 32].

This work aims to identify the developmental and genetic mechanisms responsible for petal loss in *Bocconia frutescens* and *Macleaya cordata*, having as references the typical Papaveraceae floral groundplan exhibited by *E. californica*, *P. somniferum* and *Stylophorum diphyllum*. Here we report the floral ontogeny of *B. frutescens* and *M. cordata*, and we present data on gene copy number and expression of A, B and C class gene homologs of *B. frutescens* to explain the genetic underpinning of apetaly. Finally, we also present expression data of the E class genes in the model to test how they change their typical expression patterns in the context of petal loss.

## Methods

### Plant material

Material for SEM studies was collected in FAA or EtOH 70 % from the following taxa: *Bocconia frutescens* (voucher: Colombia, Antioquia, Llanos de Cuivá, March 05, 2013, *N. Pabón*-*Mora and C. Arango*-*Ocampo292*, HUA); *Macleaya cordata* (voucher: USA, New York, Bronx, Lehman College, Davis Hall, April 09, 2011*N. Pabón*-*Mora 250,* NY); and *Stylophorum diphyllum* (voucher: USA, New York, Bronx, Living collections, New York Botanical Garden, April 16, 2011, *N. Pabón*-*Mora254*, NY).

Material for gene expression studies was collected in liquid nitrogen directly from field collections from *Bocconia frutescens* (voucher: Colombia, Antioquia, Rionegro, August 1, 2014, *N. Pabón*-*Mora and C. Arango*-*Ocampo337*, HUA). Material was collected separately from floral buds, anthetic flowers, fruits and leaves. Floral buds were collected in four different developmental stages: The first (S0) is characterized by having stigmas inserted into the green sepals, the second (S1) is characterized by having slightly exerted stigmas through the green sepals, the third (S2) is characterized by having fully exerted, non-rotated stigmas over the green sepals, and the fourth (S3) is characterized by having fully exerted rotated (90°) stigmas over the pink sepals. For stages S1, S2, S3 floral organs were also dissected into sepals, stamens and carpels. Fruits were separated into young green fruits (Fr1) and mature yellow fruits (Fr2) (Figs. 1e–h, 8a–i). Biological replicates of these collections were made to corroborate gene expression via qRT-PCR.

### Morphological studies

**Fig. 2:**
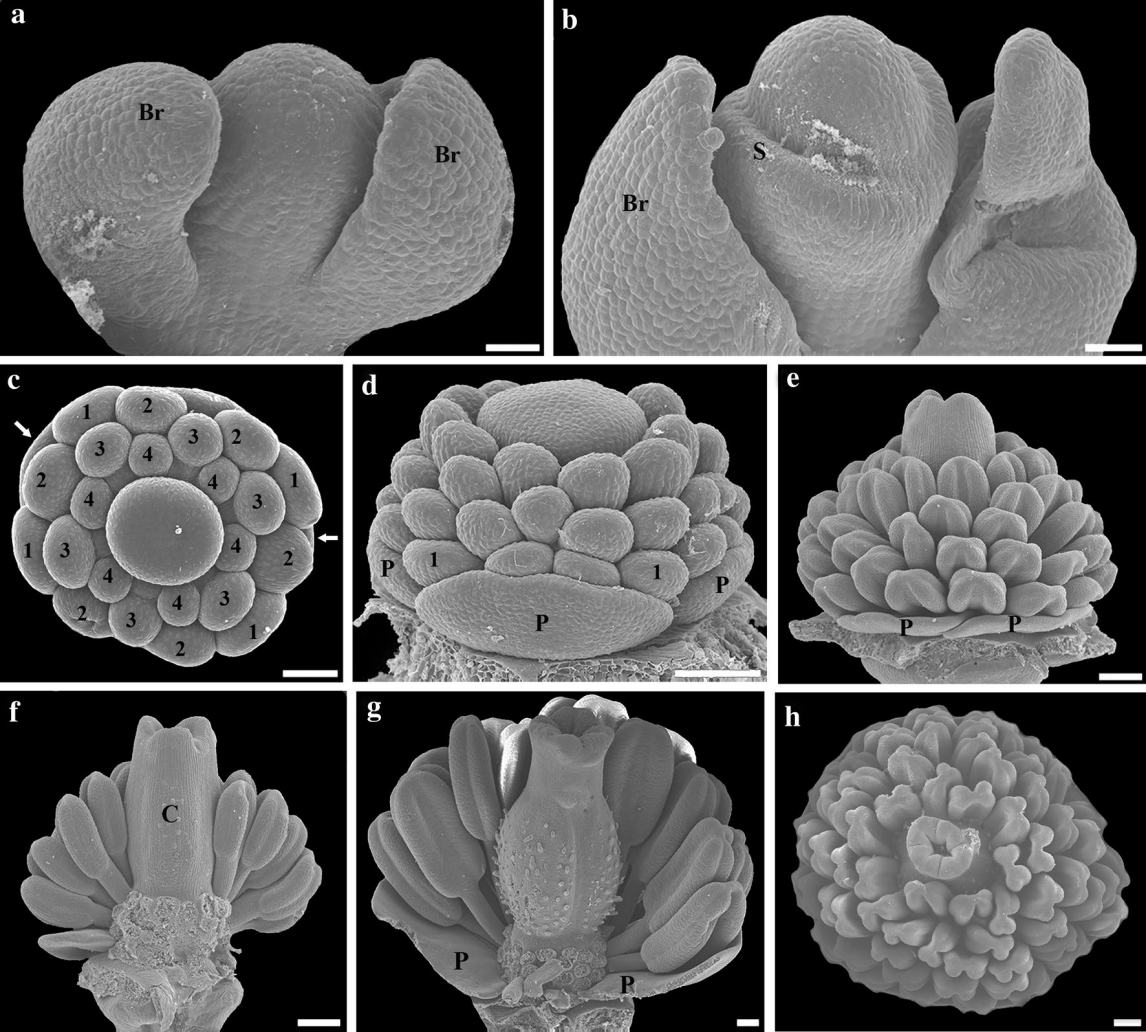
Scanning electron micrographs of floral development of *Stylophorum diphyllum*. **a** Flower primordium. **b** Sepal initiation. **c**, **d** Initiation of petals (*arrows* in **c**), stamens (numbered) and gynoecium. **e**–**h** Successive growth stages of stamens and gynoecium; some stamens removed in **f** and **g**. *Br* bract, *C* carpel, *P* petal, *S* sepal; 1, 2, first and second sets of the first whorl; 3, stamens of the second whorl; 4, stamens of the third whorl. *Bars* **a**–**c** = 50 µm; **d**–**h** = 100 µm

**Fig. 3:**
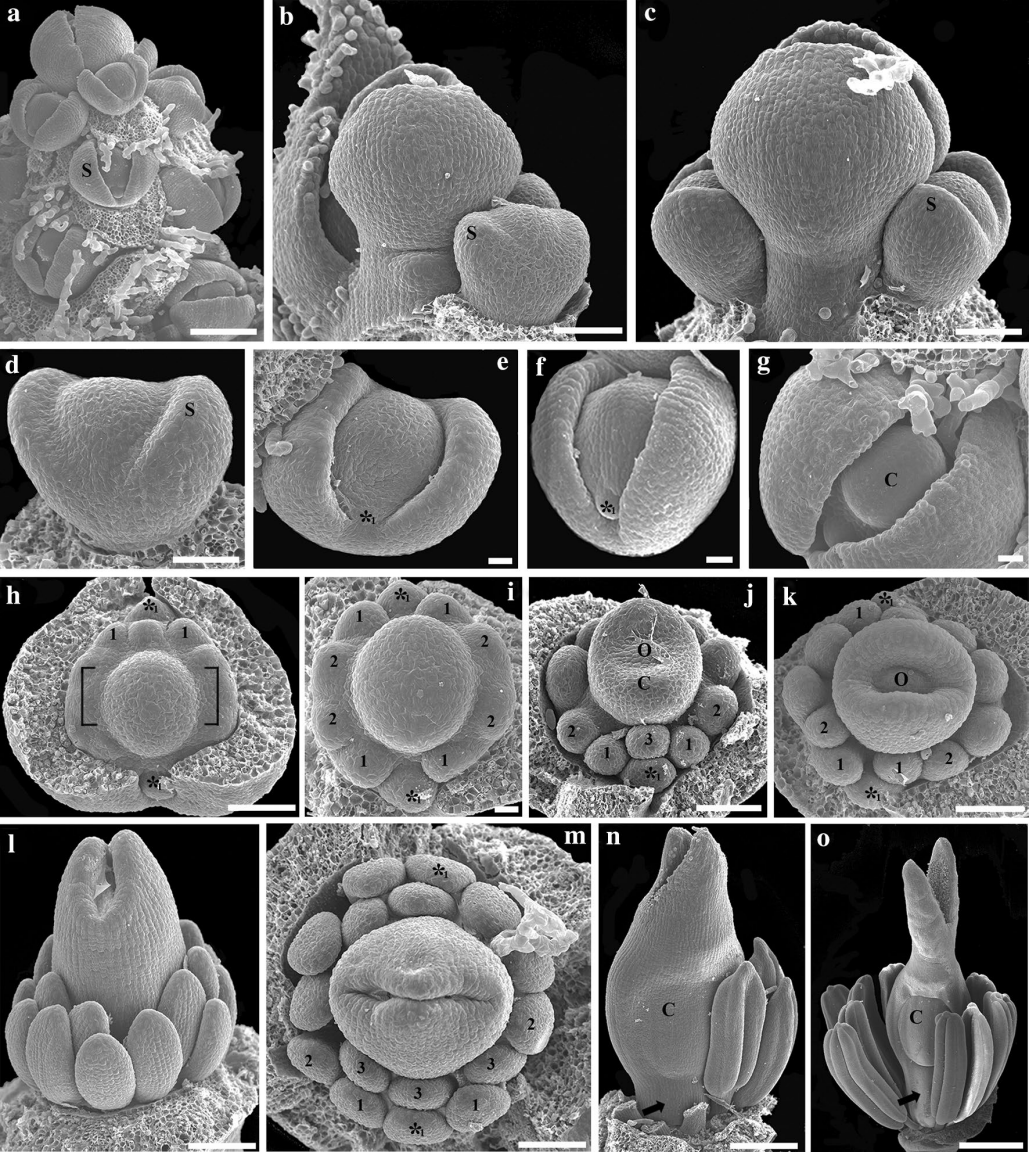
Scanning electron micrographs of floral development of *Bocconia frutescens*. **a** Developing inflorescence. **b**, **c** Detail of the apex of the inflorescence; note the larger terminal flower with respect to the two lateral flowers. **d** Initiation of the sepals. **e**, **f** Initiation of the two homeotic stamens (*_1_). **h** Initiation of the first whorl of true stamens alternate to the sepals (1); note the two opposite common primordia ([ ]). **i** Initiation of the second (*inner*) set of true stamens of the first whorl, developed from common primordial. **j**–**m** Initiation of the third set of true stamens belonging to the second whorl (3) and the ovule. **n**, **o** Late development of stamens and gynoecium; note the conspicuous stigmatic tissue, the horse-shoe-shaped valve on each carpel and the gynophore (*arrow*); *C* carpel, *O* ovule, *S* sepal, * homeotic stamens; 1, first whorl of true stamens; 2, second set of true stamens belonging to the first whorl; 3, third set of true stamens belonging to the second whorl. *Bars* **a**–**d**, **h**, **j**–**o** = 50 µm; **e**–**g**, **i** = 10 µm

Vegetative and flowering shoots of *B. frutescens, M. cordata* and *S. diphyllum* (Figs. 1, 2, 3) were fixed for 48 h in formalin–acetic acid–ethanol (3.7 % formaldehyde, 5 % glacial acetic acid, 50 % ethanol) and then transferred and stored in 70 % ethanol. For SEM, buds were dissected in 90 % ethanol under a Zeiss Stemi DV4 stereoscope and then dehydrated in an absolute ethanol/acetone series. Dehydrated material was then critical point-dried using a Balzer 790 CPD (Balzer Union, Furstentum, Liechtenstein, Rockville, MD), coated with gold and palladium in a 6.2 sputter coater at the New York Botanical Garden and examined using a JEOL (Tokyo, Japan) JSM-5410 LV SEM at 10 kV, onto which digital images were stored. Field photographs were taken with a Nikon FM-2 camera.

### Transcriptome analysis

Fresh inflorescences carrying floral buds and flowers during organ initiation (Fig. 3), as well as young flowers in S0–S4 (see above in “Plant material”) and flowers in anthesis from three different cultivated plants, were mixed together and were ground using liquid nitrogen. Further total RNA extraction was carried out using TRIzol reagent (Invitrogen). The RNAseq experiment was conducted using the truseq mRNA library construction kit (Illumina) and sequenced in a HiSeq 2000 instrument reading 100 bases paired end reads. A total of 25,185,945 raw read pairs were obtained. The transcriptome was assembled *de novo*. Read cleaning was performed with prinseq-lite with a quality threshold of Q35, and contig assembly was computed using Trinity package following default settings. Contig metrics are as follows: total assembled bases: 149,710,500; total number of contigs (>101 bases): 211,821; average contig length: 706 b; largest contig: 17,004 b; contig N50: 1877 bp; contig GC%: 40.09; number of Ns: 0. Orthologous gene search was performed using BLASTN [33] with the reference sequences downloaded from GenBank (see below). Protein domain searches were carried out using HMMER and the PFAM database downloaded from the Danger Institute FTP server. Top hits for each sequence were individually analyzed and confirmed by protein domain searches, prior to phylogenetic analysis.

### Cloning and gene characterization

For each of the gene families, searches on the transcriptome were performed by using other Ranunculales sequences as a query to identify a first batch of homologs using Blast tools [33]. Query sequences for *FRUITFULL*-*like* genes came from [24]; for *APETALA3* and *PISTILLATA* genes came from [7, 15, 21, 25]; for *AGAMOUS* genes came from [18, 20]; and for *SEPALLATA* genes came from [19]. After a search for homologous genes in the *B. frutescens* transcriptome we were able to identify 3 *FUL*-*like* (*FUL*), 1 *APETALA3* (*AP3*), 4 *PISTILLATA* (*PI*), 1 *AGAMOUS* (*AG*) and 5 *SEPALLATA* (*SEP*) homologs. We named them *BofrFL1*, *BofrFL2*, *BofrFL3*, *BofrAP3*, *BofrPI1*, *BofrPI2*, *BofrPI3*, *BofrPI4*, *BofrAG*, *BofrSEP1*-*1*, *BofrSEP1*-*2*, *BofrSEP2*, *BofrSEP3*-*1* and *BofrSEP3*-*2*. Sequences are deposited in GenBank with the accession numbers: KF500160, KF500161, KX574344-KX574356.

### Phylogenetic analyses

Sequences in the transcriptome were added to each dataset consisting of sequences available from NCBI, as well as genes available in Phytozome, specifically in the *Aquilegia coerulea* genome (http://www.phytozome.net/). Other homologs for all MADS-box gene lineages studied here were isolated from the 1kp transcriptome database (http://218.188.108.77/Blast4OneKP/home.php), Phytometasyn (http://www.phytometasyn.ca) and Plantrans DB (http://lifecenter.sgst.cn/plantransdb) after a search in the available transcriptomes of members of the Ranunculales (Additional file 1: Table S1). A matrix for each gene lineage was generated. All sequences were then compiled using Bioedit (http://www.mbio.ncsu.edu/bioedit/bioedit.html), where they were cleaned to keep exclusively the open reading frame. Nucleotide sequences were then aligned using the online version of MAFFT (http://mafft.cbrc.jp/alignment/server/) [34], with a gap open penalty of 3.0, an offset value of 0.8 and all other default settings. The alignment was then refined by hand using Bioedit taking into account the protein domains and amino acid motifs that have been reported as conserved for the five gene lineages. Maximum likelihood (ML) phylogenetic analyses using the nucleotide sequences were performed in RaxML-HPC2 BlackBox [35] on the CIPRES Science Gateway [36]. The best performing evolutionary model was obtained by the Akaike information criterion, using ModelTest incorporated in MEGA6 [37]. Bootstrapping was performed according to the default criteria in RAxML where bootstrapping stopped after 200–600 replicates when the criteria were met. Trees were observed and edited using FigTree v1.4.0.

### Expression studies

For RT-PCR total RNA was prepared using TRIzol^®^ reagent (Ambion, Grand Island, NY, USA) from all of the dissected organs and stages mentioned above. Genomic DNA contamination was removed using DNaseI (RNase-free, Austin, TX, USA) following the manufacturer instructions. First-strand cDNA was synthesized from 5 µg of total RNA using the SuperScript^®^ III First-Strand Synthesis System (Invitrogen, Grand Island, NY, USA) with the oligodT_20_ primers, following the manufacturer instructions. PCRs were carried out using EconoTaq^®^ Plus (Lucigen, Middleton, USA) following the manufacturer protocol using 1 µl of cDNA as template in a total volume of 20 µl. We manually designed primers to specifically amplify each gene copy targeting sequences unique to each gene in the K and C domains ranging between 160 and 360nt (Additional file 2: Table S2). Specific primers were hard to design for the *PISTILLATA* copies as *BofrPI1* and *BofrPI3* on the one hand and *BofrPI2* and *BofrPI4* on the other, shared identical amino acid sequences with few nucleotide changes and small insertions (30nt) in *BofrPI1* and *BofrPI2*. After multiple attempts to separate double bands in agarose gels and confirmation through sequencing we were able to obtain *BofrPI1* and *BofrPI3* independent products but were not able to distinguish between *BofrPI2* and *BofrPI4*. The following PCR program was used: 2 min at 94 °C; followed by 27–32 cycles of 30 s at 94 °C, 30 s at 55 °C and 1 min at 72 °C; followed by 10 min at 72 °C. A total of 3–4 µl of each PCR product were run on a 1 % agarose gel and digitally photographed using a Transilluminator Biometra Ti5 BioDocAnalyze.

For quantitative real-time PCR (qRT-PCR) total RNA was prepared from biological replicates with the same protocols as for RT-PCR. cDNA was synthesized from 3 µg of total RNA. Selected organs for qRT-PCR included sepals, stamens and carpels, from early S1 stages, late S3 stages and anthesis as well as late developing fruits, as they represent the entire variation for gene expression detected by RT-PCR. The resulting cDNA was diluted 1:4. PCR product was amplified using locus-specific primers designed by introducing selected paralog-specific regions into online tools available at https://www.genscript.com/ssl-bin/app/primer (Additional file 2: Table S2). For the *Bocconia frutescens PISTILLATA1/3* and *PI2/4* paralogous copies specific amplification was complicated by extensive sequence similarity, so they were amplified by pairs and not individually. Quantitative PCR was performed as described in [26]. Glyceraldehyde 3-phosphate dehydrogenase (*GADPH*), *ACTIN2* (*ACT2*) and *ELONGATION FACTOR 1α* (*EF1α*) were tested as putative endogenous controls, and *GADPH* was selected as the control for gene target quantification as it showed the lowest variation of Ct values across organs. The level of the ABC homologs *BofrAP3*, *BofrPI1/3*, *BofrPI2/4*, *BofrAG*, *BofrFL2* and *BofrFL3* was analyzed relative to GADPH using the 2^−ΔΔCt^ method [38], and the relative level across organs was set to be compared to the organ with the highest expression detected per gene arbitrarily set to 1. The expression of *BofrFL1* was not examined as RT-PCR results already suggested low expression. Quantitative RT-PCRs were performed using the qTower2.2 qPCR system and the qPCRsoft software (Analytik Jena).

## Results

### Flower development of *Stylophorum diphyllum*

Flower development of *S. diphyllum* begins with the simultaneous initiation of two sepal primordia from a radial flower primordium (Fig. 2a, b). The two outer petal primordia arise simultaneously from flat primordia that alternate with the sepal primordia; then, the two inner petals initiate (Fig. 1c, d, 2c–e). The four petal primordia grow slowly during early stages of floral development (Fig. 2d,e). Flower development continues with the centripetal initiation of four early isodiametric stamen primordia arranged in an outer whorl that gets filled in with 10–12 staminal primordia, then a second whorl of 6–10 primordia that alternate with the outer ones and 5–10 primordia that form the inner whorl; sometimes, the latter ones are roughly arranged in two, instead of one (Figs. 1d; 2c–e). Mature stamens are formed by an upright filament and a short flattened anther, both yellow (Fig. 1c). Flower development ends with the formation of four fused carpels with trichomes externally and four massive commissural placentas internally (Figs. 1c,d, 2e–h).

### Flower development of *Bocconia frutescens*

Each inflorescence of *B. frutescens* consists of groups of 1–3 pedicellate flowers in which the terminal one develops first and is larger than the lateral ones (Fig. 3a–c). Floral primordia are hemispherical. Flower development begins with the initiation of two synchronous sepals (Fig. 3b–d); rarely, three sepals are formed in the terminal flower (Additional file 3: Fig. S1). Then, two simultaneous stamens initiate alternating with the sepals in the position of the first two petals (Figs. 1i, 3e, f). Thus, we consider these two stamens to be homeotic (see “Discussion”). No primordia develop opposite to the sepals, where the second whorl of petals is expected by comparison with closely related petalous species (Figs. 1i, 3h, j). Next, the initiation of 4 stamens is evident in the fourth whorl, followed by 5 or 6 additional stamens sometimes developed from a common primordium (Fig. 3h, i) opposite to the sepals (Fig. 3i). Then, a second whorl of 3–7 stamens develops alternating with the previous one (Fig. 3j–m). The number and arrangement of the stamens are variable, but they appear to occur in whorls rather than in spiral, although the second whorl of stamens often possesses less stamens in comparison with the first one (Fig. 3j–m). The number of stamens is often higher in the terminal flowers reaching up to 21 (Additional file 3: Fig. S1). The two carpel primordia develop next subtended by a gynophore that grows conspicuously during preanthesis (Fig. 3j–o). Closure of the carpels occurs late, accompanied by the formation of the dehiscent horse-shoe-shaped valve (Fig. 3m–o). In addition, the style and the stigma grow and differentiate conspicuously. Carpels at preanthesis are three times the total length of the stamens (Fig. 3l). Each mature stamen consists of a filiform filament and an elongated anther that occupies the 2/3-s distal-most portion of the stamen (Fig. 1g, h). Final changes during preanthesis include the protrusion of the stigmas through the sepals, the orientation of the stigmatic lobes toward the carpel commissures and the change in the color of the sepals from green to pink (Fig. 1e, f). During anthesis the sepals are caducous, and all stamens (both homeotic and true) are pendulous and produce copious pollen (Fig. 1g, h).

### Flower development of *Macleaya cordata*

**Fig. 4:**
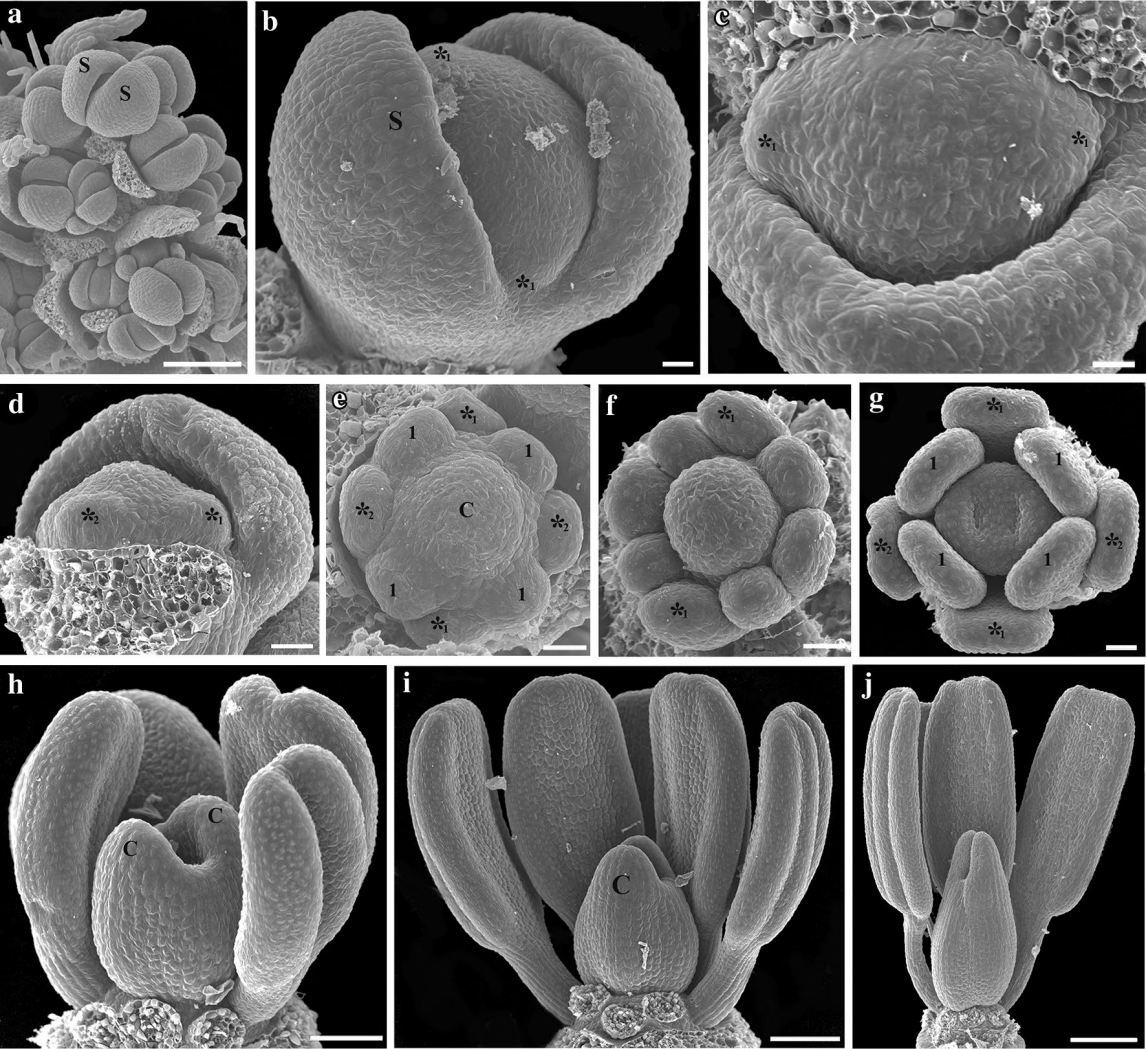
Scanning electron micrographs of floral development of *Macleaya cordata.* **a** Developing inflorescence; note the larger terminal flower. **b** Initiation of sepals. **c**, **d** Initiation of the first whorl of homeotic stamens, alternate to the sepals (*_1_). **e**–**g** Successive initiation of the second whorl of homeotic stamens, opposite to the sepals (*_2_), and the single whorl of four, decussate true stamens (1). **h**–**j** Growth of the stamens and the short, sessile gynoecium. *C* Carpel, *S* sepal, * homeotic stamens; 1, first whorl of true stamens. *Bars* **a**, **j** = 100 µm; **b**–**g** = 10 µm; **h**, **i** = 50 µm

The inflorescence of *M. cordata* consists of groups of 1–3 pedicellate flowers in which the terminal one develops first (Fig. 4a). The floral primordium is hemispherical in shape (Fig. 4a); the primordium of the terminal flower is often larger than that of the lateral flowers; however, unlike *B. frutescens*, flower development of the terminal and the lateral flowers in *M. cordata* is identical. Flower development begins with the initiation of two synchronous sepals (Fig. 4b). This is followed by the initiation of the four homeotic stamens in two decussate whorls; the first whorl is formed alternating with the sepals, and the second whorl is formed opposite to the sepals in the positions where petals are expected to occur (Figs. 1m, 4b–e). True stamens (4) initiate next in a single whorl (Fig. 4e–g), and unlike *B. frutescens*, these are the only stamens formed (Fig. 1m). Thus, *M. cordata* flowers consistently have a total of eight stamens. Flower development ends with the differentiation of a two carpellate gynoecium, which, unlike that of *B. frutescens*, lacks a gynophore (Fig. 4g–j). Closure of the two carpels occurs late in development, and no tissue differentiation occurs between valves and commissural tissue (Fig. 4j). At preanthesis, carpels reach less than half of the total length of the stamens (Fig. 4i). Final changes during preanthesis include the change of the color of the sepals from green to yellow (Fig. 1j–m). During anthesis the sepals are caducous, and the stamens are pendulous (Fig. 1l).

### Evolution of the *APETALA3* and *PISTILLATA* gene lineages in Ranunculales

**Fig. 5:**
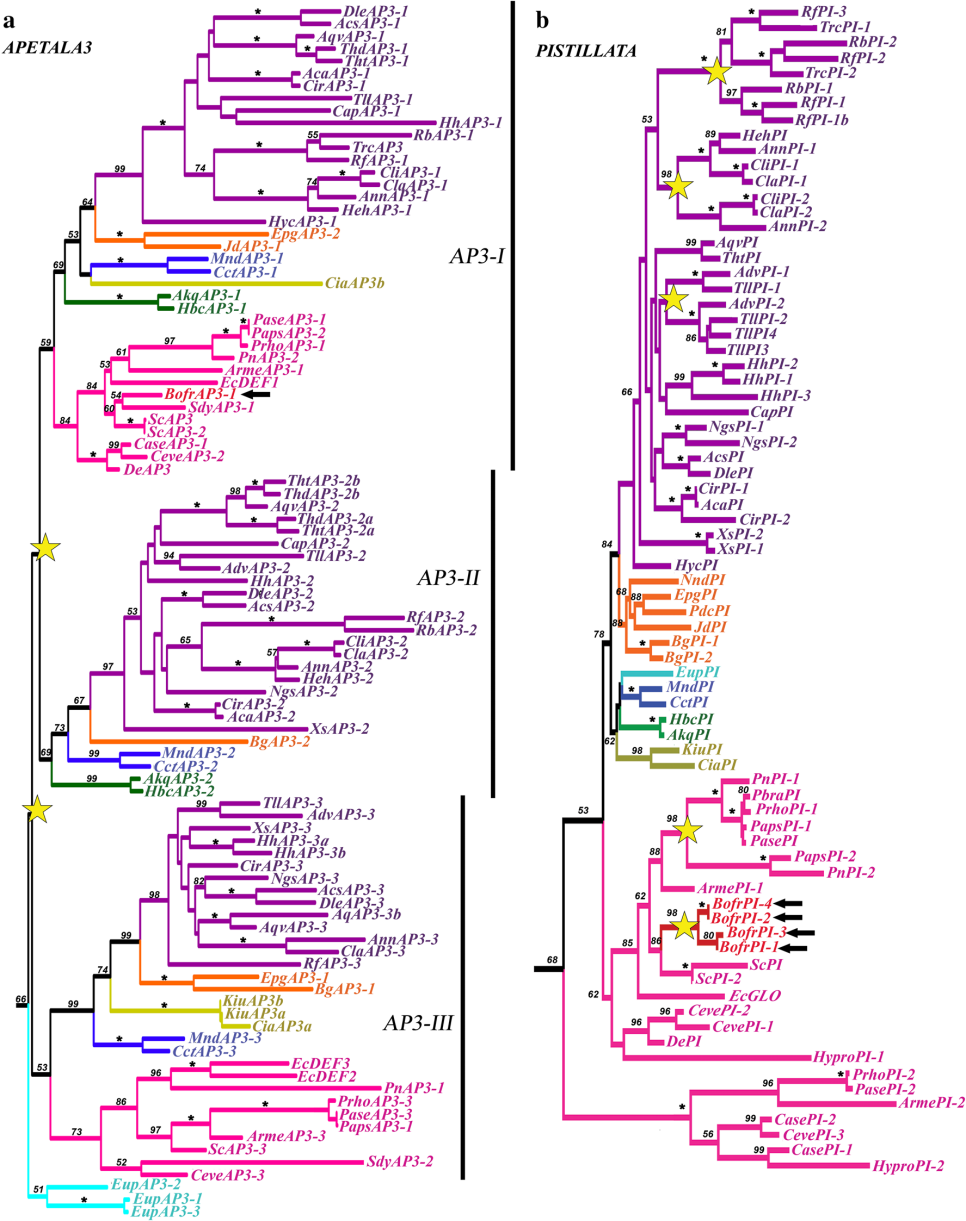
Maximum likelihood trees of *APETALA3* (**a**) and *PISTILLATA* (**b**) in Ranunculales. *Colors* indicate the following taxa: *light blue* Eupteleaceae; *pink* Papaveraceae; *green* Lardizabalaceae; *straw* Circaeasteraceae; *dark blue* Menispermaceae; *orange* Berberidaceae; *purple* Ranunculaceae. *Stars* indicate gene duplications. *Arrows* point to the single copy of *AP3* (*BofrAP3-1* in **a**) and the four *PI* paralogs (*BofrPI1*/*BofrPI2*/*BofrPI3*/*BofrPI4* in **b**) in *B. frutescens*. Bootstrap values (BS) above 50 are placed at nodes; *asterisks* indicate BS of 100

The maximum likelihood (ML) *AP3* analysis recovered three deeply conserved, paralogous lineages, termed *AP3*-I, *AP3*-*II* and *AP3*-*III* in the Ranunculales. The *AP3*-*III* clade is sister to *AP3*-*I* and *AP3*-*II* and has been functionally assigned to a petal-specific role. The Papaveraceae possesses gene members only in the *AP3*-*I* and *AP3*-*III* clades; so far, *AP3*-*II* genes have not been recovered from any Papaveraceae species [7, 25, 39] (Fig. 5a). Sampling within Papaveraceae has been scarce, especially in the Chelidonieae, and *AP3* genes have been found only in *Bocconia frutescens,Sanguinaria canadensis* and *Stylophorum diphyllum* (Fig. 5a). Unlike in other closely related petalous species, the *AP3*-*III* putative copy was not found in the floral transcriptome of *B. frutescens*, which suggests that *AP3*-*III* is not expressed. The remaining *AP3*-*I* ortholog, named *BofrAP3*-*I,* is expressed predominantly in stamens, carpels and fruits. In addition, *BofrAP3* is turned on in sepals when they switch color from green to pink (Fig. 8j, k).

The *PISTILLATA* ML analysis shows four *PI* duplicates unique to *B. frutescens,* namely, *BofrPI*-*1, BofrPI*-*2, BofrPI*-*3 and BofrPI*-*4* (Fig. 5b). Taxon-specific duplications seem to have occurred also in other species, such as *Berberis gilgiana* (Berberidaceae), and the Ranunculaceae *Xantorrhiza simplicissima*, *Helleborus hybrida* and species of *Adonis, Anemone, Ranunculus*, *Thalictrum* and *Trollium* (Fig. 5b) [21]. Results on gene expression of the four *BofrPI* orthologs are mainly restricted to stamens and only sometimes extend to sepals (*BofrPI1, BofrPI3*), carpels (*BofrPI3*) and leaves (*BofrPI2/4*) (Fig. 8j, l, m). *BofrPI1* and *BofrPI2/4* have opposite expression levels, as stamens grow older, while *BofrPI1* increases, *BofrPI2/4* levels decrease (Fig. 8j).

### Evolution of the *APETALA1/FRUITFULL* gene lineage in Ranunculales

**Fig. 6:**
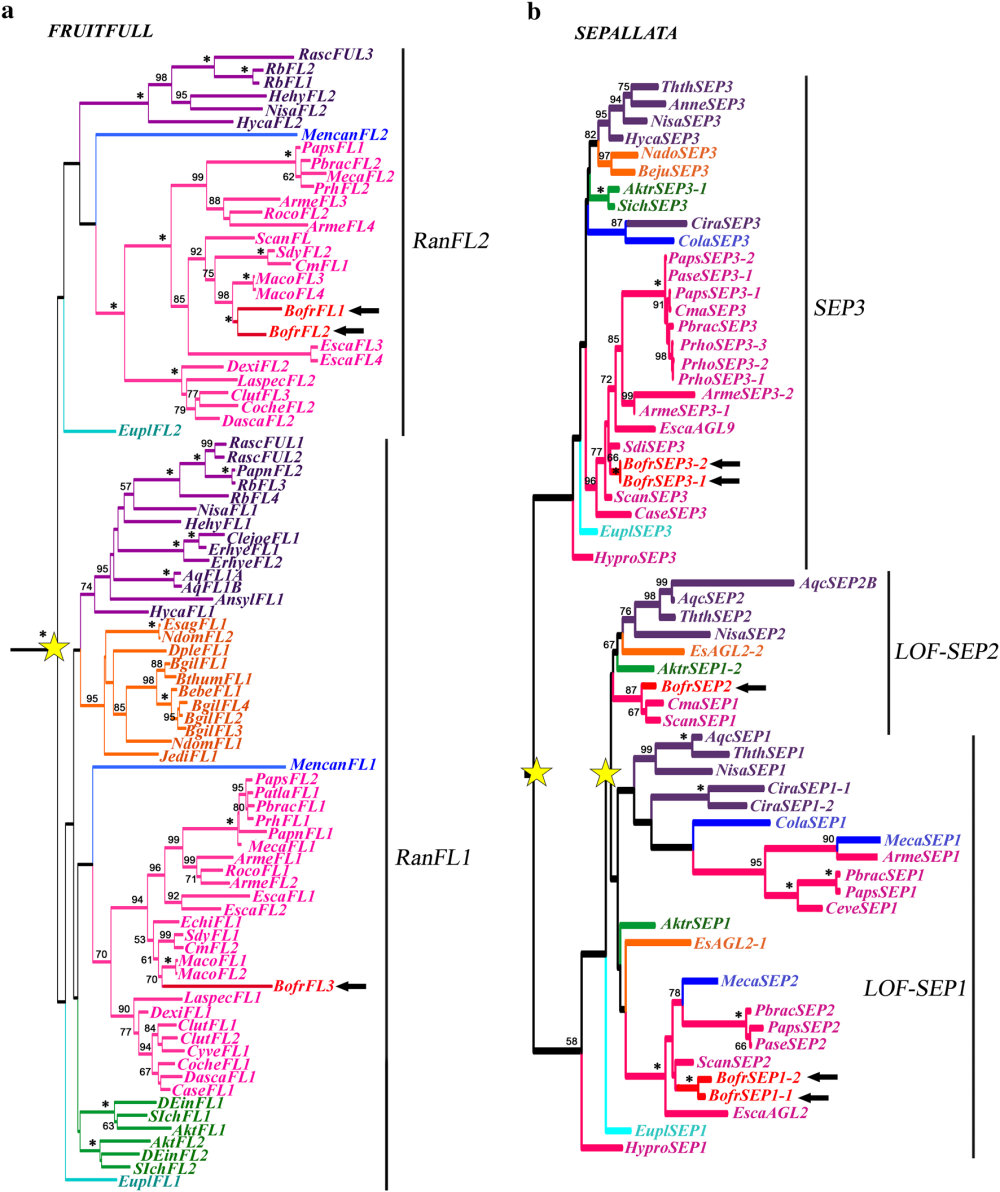
Maximum likelihood trees of *FUL*-*like* (**a**) and *SEPALLATA* (**b**) in Ranunculales. *Colors* indicate the following taxa: *light blue* Eupteleaceae; *pink* Papaveraceae; *green* Lardizabalaceae; *dark blue* Menispermaceae; *orange* Berberidaceae; *purple* Ranunculaceae. *Stars* indicate gene duplications. *Arrows* point to three *FUL*-*like* paralogs (*BofrFL1*/*BofrFL2*/*BofrFL3* in **a**) and five *SEP* copies (*BofrSEP1*-*1*, *BofrSEP1*-*2*, *BofrSEP2*, *BofrSEP3*-*1* and *BofrSEP3*-*2* in **b**) in *B. frutescens*. Bootstrap values (BS) above 50 are placed at nodes; *asterisks* indicate BS of 100

ML analysis of the *AP1/FUL* genes recovered a single duplication early in the diversification of the Ranunculales resulting in two clades of *FUL*-*like* genes, named *RanFL1* and *RanFL2* [24]. Bootstrap support for the *RanFL1* and *RanFL2* clades is low (>50); however, gene copies from the same family within each clade are grouped together with strong support, and the relationships among gene clades are mostly consistent with the phylogenetic relationships of the sampled taxa [8]. The Papaveraceae have representative genes in both clades. All members of Chelidonieae have two copies, except for the taxa-specific duplications in *M. cordata* and *B. frutescens,* which have four and three copies, respectively. *MacoFL1* and *MacoFL2* are orthologs of *BofrFL3* in the *RanFL1* clade, whereas *MacoFL3* and *MacoFL4* are orthologs of *BofrFL1* and *BofrFL2* in the *RanFL2* clade (Fig. 6a). Expression patterns for *FUL*-*like* homologs are variable. *BofrFL3* is broadly expressed in sepals, stamens, carpels and fruits, *BofruFL2* is mostly restricted to sepals and carpels, and finally *BofrFL1* is expressed at very low levels predominantly in sepals, stamens and leaves (Fig. 8j,o,p).

### Evolution of the *SEPALLATA* gene lineage in Ranunculales

The ML analysis of *SEP* genes shows three duplication events in the diversification of the Ranunculales, resulting in three clades, the *SEP3* clade and two *LOF*-*SEP* clades (*LOF*-*SEP1* and *LOF*-*SEP1*) (Fig. 6b). *B. frutescens* has the following five paralogs: *BofrSEP1*-*1* and *BofrSEP1*-*2*, which belong to the *LOF*-*SEP1* clade; *BofrSEP2*, which belongs the *LOF*-*SEP2* clade; and *BofrSEP3*-*1* and *BofrSEP3*-*2*, from the *SEP3* clade. *BofrSEP2* has a broad expression in all floral organs and fruits in every developmental stage. *BofrSEP1*-*1* and *BofrSEP1*-*2* have overlapping expression in stamens and carpels, but the expression of *BofrSEP1*-*1* extends to sepals, fruits and leaves (Fig. 8j). On the other hand, *BofrSEP3*-*1* and *BofrSEP3*-*2* are expressed in stamens, carpels, fruits and leaves, but while *BofrSEP3*-*2* is strongly expressed, the levels of *BofrSEP3*-*1* are almost undetected (Fig. 8j).

### Evolution of the *AGAMOUS* gene lineage in Ranunculales

**Fig. 7:**
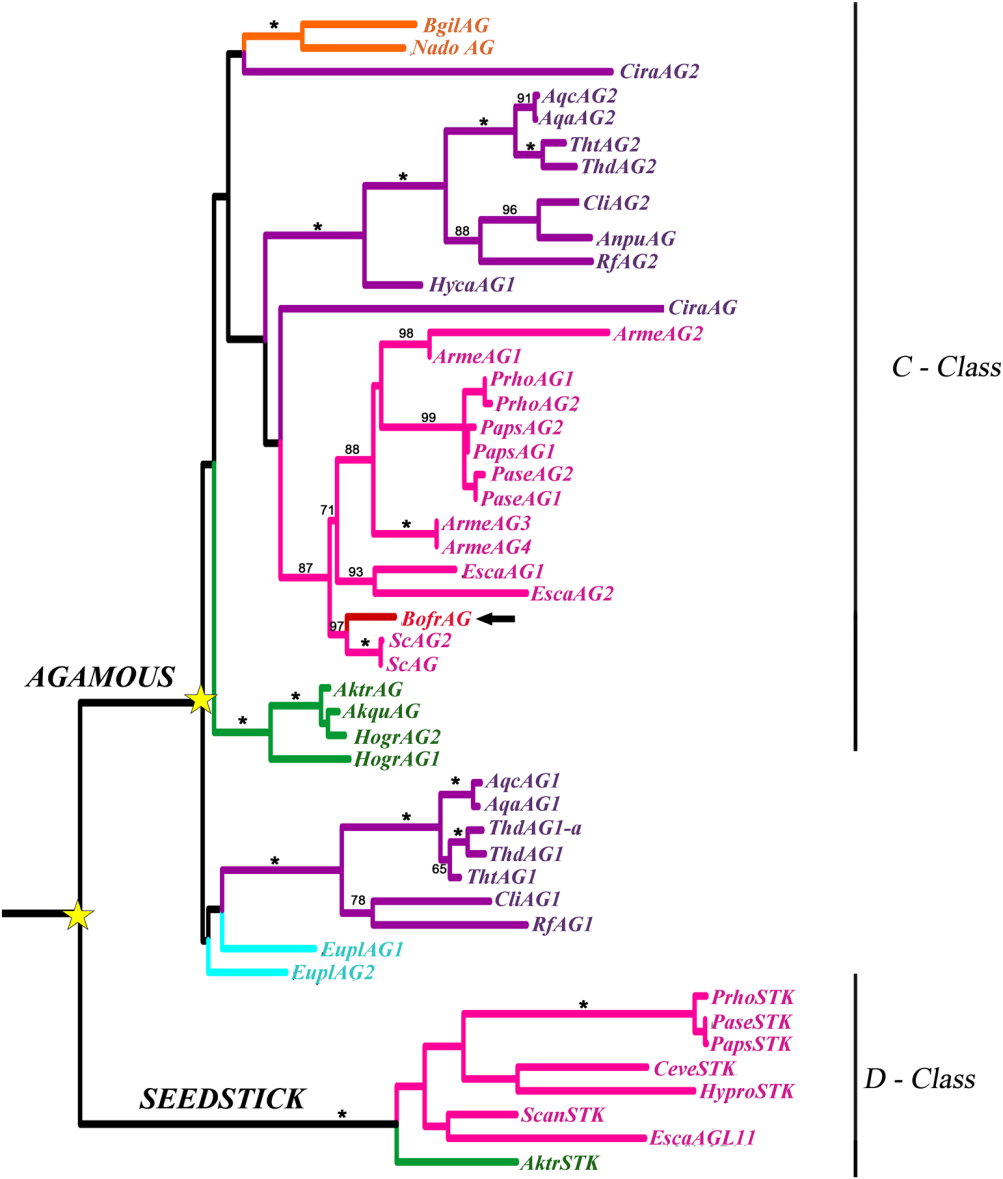
Maximum likelihood tree of *AGAMOUS* in Ranunculales. *Colors* indicate the following taxa: *light blue* Eupteleaceae; *pink* Papaveraceae; *green* Lardizabalaceae; *dark blue* Menispermaceae; *orange* Berberidaceae; *purple* Ranunculaceae. *Stars* indicate gene duplications. *Arrow* points to the single copy of *AG* (*BofrAG*) in *B. frutescens*. Bootstrap values (BS) above 50 are placed at nodes; *asterisks* indicate BS of 100

**Fig. 8:**
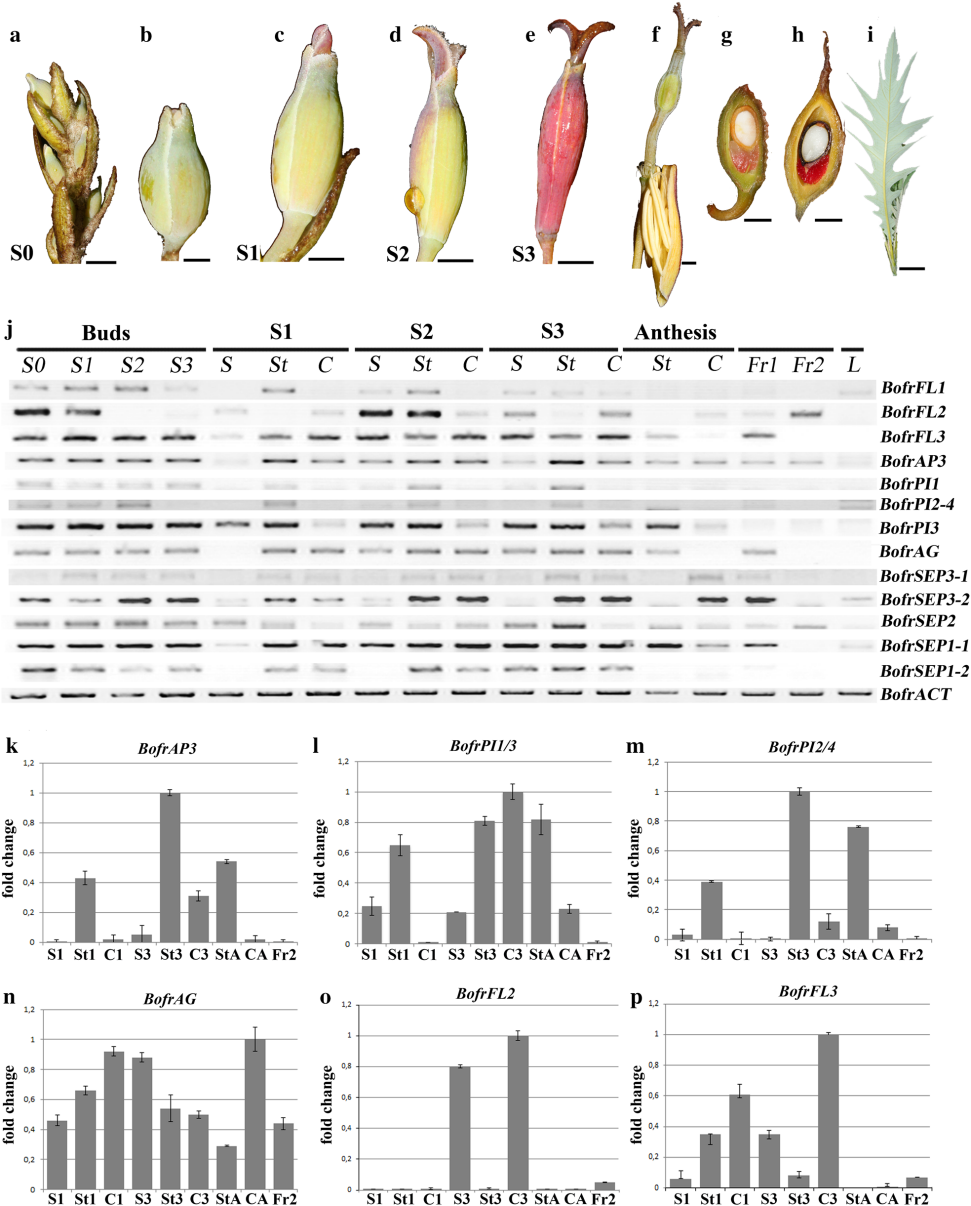
Floral stages and dissected organs of *B. frutescens* with corresponding expression studies. Inflorescence (**a**) and floral bud (**b**) at Stage 0 (S0). Floral buds at stages S1 (**c**), S2 (**d**) and S3 (**e**). **f** Flowers at anthesis. Young (Fr1) (**g**) and mature fruits (Fr2) (**h**). **i** Leaves. **j** Locus-specific RT-PCR results for all isolated MADS-box genes. Sepals (S), stamens (St) and carpels (C) were also dissected individually at stages S1, S2, S3. *ACTIN* (*BofrACT*) was used as a positive control. *L* Leaf. Numbers after S and Fr correspond to the different developmental stages. Relative expression patterns of *BofrAP3* (**k**), *BofrPI1/3* (**l**), *BofrPI2/4* (**m**), *BofrAG* (**n**), *BofrFL2* (**o**), *BofrFL3* (**p**) in selected floral organs. Values are mean ± SD for three technical replicates. *Bars* in **a**–**i** = 0.5 mm

The ML reconstruction for *AG* genes exhibits a large-scale duplication previous (at least) to the diversification of the Ranunculaceae as well as numerous species-specific duplications in the Papaveraceae (Fig. 7). However, a single copy of *AG* was found in *B. frutescens*. *BofrAG1* is expressed predominantly in stamens and carpels in all stages of flower development, but an extended expression was detected in sepals during S1, S2 and S3 and in young fruits (Fig. 8j, n).

## Discussion

Early floral development in *Bocconia frutescens,Macleaya cordata* and *Stylophorum diphyllum* is strikingly similar to all species of Papaveraceae studied to date (i.e., *Chelidonium maju*s Lour., *Eschscholzia californica, Papaver somniferum* and *Sanguinaria candensis*), as they initiate from an elliptic or hemispheric floral apex, from which sepals form first (Figs. 2a,b, 3a–e, 4a,b) [10, 28, 40–42]. Most Papaveraceae develop an outer and an inner whorl of two petals; the two outer petals develop first alternating with the two sepals, followed by the two inner petal primordia (Figs. 1d, 2c–e) [10, 28, 40–42]. Although both *B. frutescens* and *M. cordata* exhibit a petal-to-stamen homeosis, as stamens develop in the place and time of petals compared to petalous closely related species resulting in apetalous flowers, they differ in the number of homeotic staminal whorls that actually develop. In *B. frutescens* only the outer whorl of petals is clearly replaced by homeotic stamens, and the second one, opposite to the sepals, does not develop (Figs. 1i, 3g–k), whereas in *M. cordata* both whorls of petals are replaced by homeotic stamens (Figs. 1m, 4d–g). Homeotic stamens in *B. frutescens* and *M. cordata* are bulged and rounded from early stages of development, suggesting complete homeosis (Figs. 3f, 4d,e). These results contrast with the stamen-to-petal homeosis observed in *S. canadensis*, which exhibits flowers with eight petals. In *S. canadensis* both the stamen primordia and the homeotic petal primordia are bulged and rounded, contrasting with the true petals that are broad compared to their length [10]. In turn, early ontogeny of the homeotic petals differs from that of true petals, and homeosis occurs late in development. This shows that homeosis can vary in terms of placement and timing in different Papaveraceae species.

The number of staminal whorls varies across Papaveraceae from one in *M. cordata* to two in *B. frutescens*, three in *E. californica* (and the terminal flowers of *B. frutescens;* Additional file 3: Fig. S1), four in *S. canadensis*, six in *C. majus* and *S. diphyllum* or many in *P. somniferum* (Figs. 2c,d, 3i–k, 4e–g) [10, 40–42]. Such variation suggests a high degree of developmental flexibility in the construction of staminal whorls, as it was noticed by Sattler [42], who also pointed out that irregularities are rather common and the notion of defining whorls is an over simplification in Papaveraceae. The number of stamens per whorl also varies dramatically in different species and even intraspecifically in flowers occupying different positions in the inflorescence (Figs. 2c–e, 3j, k, m; Additional file 3: Fig. S1). However, homeotic stamens of the first whorl are exceptional in that they are always paired and occupy the same position as petals (Figs. 1d, i, m, 2c, 3i, 4e, g) [40, 42]. Unlike the erect and thickened stamens in most Papaveraceae (e.g., Figs. 1a–c, 2f, g), *B. frutescens* and *M. cordata* possess pendulous and versatile stamens during anthesis due to the long and slender filaments (Figs. 1g, h, l, 3o, 4j). Typically, all Papaveraceae species have two carpels with the exception of *S. diphyllum* that has four (Fig. 2g) [43] and some *Papaver* species that have up to 12 [28, 44]. Features unique for *B. frutescens* include the gynophore that pushes the gynoecium outside of the sepals during preanthesis and the papillose stigma (Figs. 3n, o, 8c–h). These traits are linked to wind pollination in other Ranunculales like *Euptelea pleiosperma* Hook. f. & Thomson and *Thalictrum* spp. and are thought to have occurred as a result of convergent evolution [45–47]. Lastly, in *B. frutescens* and *M. cordata* a single ovule develops, an uncommon feature in the family likely resulting from an evolutionary reduction (Fig. 3k) [10, 48]; the persistent red aril on the single seed of *B. frutescens* (Fig. 8c–h), a trait likely related to bird dispersal, is also unique to this genus.

### Loss of petals is likely due to *APETALA3-III* loss of function concomitant with the expansion of *AGAMOUS* expression domains in *B. frutescens*

The study of gene lineage evolution linked to expression study analyses is a plausible way to understand the acquisition and loss of floral organs; this is particularly true for genes that control petal identity [17, 21, 49]. It is known that the *AP3* gene lineage has undergone functional diversification associated with several duplications occurring before the diversification of the Ranunculales. As a result of two consecutive duplications, all members of the order have gene representatives from three clades, named *AP3*-*I, AP3*-*II* and *AP3*-*III* (Fig. 5a) [7, 21, 25]. The Papaveraceae seem to have lost the *AP3*-*II* clade paralogs and have genes only belonging to the *AP3*-*I* and the *AP3*-*III* clades [25]. Functional characterization of gene copies in *A. coerulea* (Ranunculaceae) shows that *AqAP3*-*1* specifies the identity of the staminodia, *AqAP3*-*2* controls the identity of stamens, and *AqAP3*-*3* controls petal identity. Functional data in other members of the Ranunculaceae, like *Nigella damascena,* show that while *NdAP3*-*3* has specialized in providing petal identity, *NdAP3*-*1* and *NdAP3*-*2* are functioning mostly in stamen identity and only in a dosage-dependent manner in petal shape [30, 32]. Functional data are also similar in Papaveraceae. In *Papaver somniferum* it has been shown that *PapsAP3*-*1* (belonging to the *AP3*-*III* clade) specifies petal identity, whereas *PapsAP3*-*2* (belonging to the *AP3*-*I* clade) controls stamen identity (Fig. 5a) [25, 28–30]. In general, these studies demonstrate that the three gene clades have undergone subfunctionalization, in particular the *AP3*-*I* and *AP3*-*III* clades, which have specialized in regulating stamen and petal identity, respectively. Subfunctionalization likely occurred first; thus, petal loss can occur without affecting stamen identity, as it occurs in *B. frutescens.* Our results show that the single copy expressed, *BofrAP3*, belonging to the *AP3*-*I* clade is predominantly found in stamens, consistent with functional analysis of *AP3*-*I* orthologs in Papaveraceae and Ranunculaceae (Fig. 5a, 8j) [25, 28, 29]. It was not possible to isolate any *AP3*-*III* paralog from the *B. frutescens* floral transcriptome; thus, we propose that this copy is no longer expressed, and it is linked to petal loss in *B. frutescens*. Without this *AP3*-*III* paralog, it is likely that the petal developmental program, which included an obligate *PI* heterodimer and the *AP1/FUL*-*like* or the *AGL6* homolog, is no longer active.

**Fig. 9:**
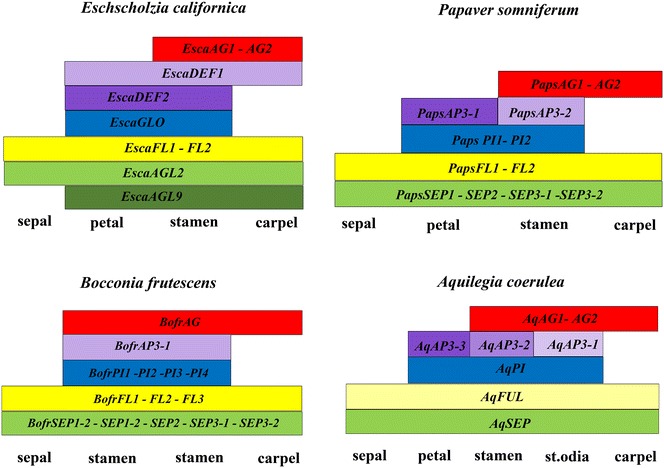
Representative scheme of the A, B, C and E-class gene expression in petalous (*Eschscholzia* *californica* and *Papaver* *somniferum*) and apetalous (*Bocconia* *frutescens*) Papaveraceae members in comparison with the model Ranunculaceae (*Aquilegia* *coerulea*). *Light yellow* in *Aquilegia* for homologs of *FUL*-*like* genes indicates broad expression in floral organs but no known roles in floral organ identity [27]. The models are taken directly or constructed based on data from Drea et al. [28], Yellina et al. [54], Pabón-Mora et al. [26, 27], Sharma and Kramer [29], Lange et al. [55]. *St.odia* Staminodia

A relation between loss of gene expression and petal loss has also been observed in a number of Ranunculaceae species, where gene loss, deletion of exonic sequences, deletion of regulatory sequences and pseudogenization of the *AP3*-*III* ortholog in different apetalous species have been shown to occur [32, 39]. Our analysis shows that the same is likely true for Papaveraceae as the *AP3*-*III* paralog is found in all Papaveraceae with petals with available transcriptomes, including the closely petalous relatives like *Sanguinaria Canadensis* and *Stylophorum diphyllum*, but the ortholog is not expressed in *B. frutescens*. One additional point to consider is that the phenotype associated with the absence of *AP3* as predicted by the ABCE model, and as it has been shown to occur after *PsomAP3*-*1* (in the AP3-3 clade) downregulation in poppies is the acquisition of sepal identity in the second whorl organs [12, 28]; however, the floral bauplan in *B. frutescens,* like those exhibited by apetalous Ranunculaceae species, results in the formation of stamens, and not sepals, in the second whorl, which is indicative of an accompanying ectopic expression of C class *AGAMOUS* genes. *AG* genes have retained their roles in stamen and carpel identity, despite having undergone duplications in core, basal eudicots and monocots [18]. *PaleoAG* preduplication genes in basal eudicots (sensu Pabón.Mora et al. [20]) have been characterized in *E. californica* and *P. somniferum* (Papaveraceae) and in *Aquilegiacoerulea, Nigella damascena* and *Thalictrum thalictroides* (Ranunculaceae), where they are responsible for stamen and carpel identity and at least in one case (*ThtAG2* from *T*. *thalictroides*) they play key roles in ovule identity [30, 50–54]. Interestingly, different *AG* copies in Ranunculales do not share a common origin. *AG* paralogs in Ranunculaceae are the result of a family-level duplication, while *AG* paralogs in *E. californica* are the result of a species-specific duplication and *AG* transcripts in *P. somniferum* are the result of alternative splicing producing two proteins with different lengths of C-terminus [20, 50–54]. Moreover, it has been shown that *E. californicaAG* copies can negatively regulate *AP3*-*3* paralogs, but not *AP3*-*1* paralogs, suggesting specific repressive interactions of C over B class genes [54]. However, it is not clear whether they are mutually repressive, as functional analysis of *AP3* homologs has not been done in *E. californica* and *AG* expression was not tested in *P. somniferum* plants showing *AP3* downregulation [28, 54]. We found that *B. frutescens* has a single copy of *AG* that is expressed predominantly in stamens and carpels in mature flowers, but also has an extended expression in sepals; therefore, we propose that concomitant with the absence of *BofrAP3*-*3*, *BofrAG* has expanded into the outer whorls (Fig. 9). In the second whorl *BofrAG* together with *BofrAP3* is likely to provide stamen identity (Figs. 7, 8j, k, n). Whether ectopic expression of *BofrAG* occurred prior to or after loss of expression of *BofrAP3*-*3* is unclear at this point and requires a deep understanding of the regulatory interactions among these key regulatory transcription factors.

### Petal loss in *B. frutescens* occurs independently of duplications and functional evolution of *PISTILLATA* genes

Contrary to the *AP3* gene lineage, the *PI* gene lineage exhibits numerous recent taxon-specific duplications resulting in exclusive paralogs in different species of Papaveraceae, Berberidaceae and Ranunculaceae (Fig. 5b). Functional studies of *PI* genes have been done in *E. californica* and *P. somniferum* (Papaveraceae) and *A. coerulea* and *Thalictrumthalictroides* (Ranunculaceae). In *E. californica* the *seirena*-*1* (*sei*-*1*) mutant showing homeotic conversions of petals to sepals and stamens to carpels was mapped to a mutation in *EcGLO,* the only *PI* ortholog in this species (Fig. 5b) [55]. The same phenotype was found after downregulation of *AqvPI* and *ThtPI* in *Aquilegia* and *Thalictrum*, respectively [31, 56]. In *P. somniferum,* a local duplication has resulted in two copies *PapsPI*-*1* and *PapsPI*-*2.* Functional characterization shows that both copies redundantly control petal and stamen identity [28]. Altogether, these data suggest that, unlike *AP3* genes, the role of *PI* genes has remained conserved over evolutionary time, and that after local duplications, paralogs retain redundancy [21]. Our sampling in *B. frutescens* detected four *PI* copies with different expression patterns, three of them (*BofrPI1, BofrPI2* and *BofrPI4*) expressed mainly in stamens, as expected; the fourth copy, *BofrPI3,* is expressed in stamens, sepals and carpels. Expression detected in the carpel can be the result of the characteristic expression of *PI* in the growing ovules [56, 57]. These data suggest that *BofrPI3* is differentially regulated compared to its paralogs and could be playing additional roles besides stamen identity in *B. frutescens* (Fig. 8j, l, m).

### *FUL-like* genes: pleiotropic genes with an uncertain contribution to petal identity

In addition to *B* class genes, the *A* class genes (*APETALA1* an *APETALA2*) are also involved in petal identity in *Arabidopsis thaliana* [12]. Numerous duplications have also occurred in these gene lineages [16, 23]. *FUL*-*like* genes in Papaveraceae are involved in floral meristem and perianth identity, transition to the reproductive meristem, cauline leaf development, branching and fruit development [26]. Most Chelidonieae have two *FUL*-*like* copies, one in each Ranunculid clade (*RanFL1* and *RanfL2*); exceptions are *Sanguinaria canadensis*, which has only one gene, and *B. frutescens* and *M. cordata*, which have three (*BofrFL1, BofrFL2* and *BofrFL3*) and four copies (*MacoFL1, MacoFL2, MacoFL3* and *MacoFL4*), respectively, as a result of species-specific duplications (Fig. 6a). In *B. frutescens*, *BofrFL2* and *BofrFL3* are broadly expressed in sepals, stamens, carpels and fruits, which suggests that they both have conserved the pleiotropic roles of other *FUL*-*like* genes in Papaveraceae [26]. Conversely, *BofrFL1* has a low expression, restricted to stamens, organs in which *FUL*-*like* genes are not known to play any developmental role. This suggests a rather different functional evolutionary path for *BofrFL1* toward loss of function through pseudogenization [58, 59].

On the other hand, *APETALA2/TOE3* genes have also diversified in angiosperms with most duplications occurring in the Brassicales, the Monocots and a local duplication in the Ranunculales [23]. *B. frutescens* possesses two *euAP2* genes named *BofrAP2* and *BofrAP2*-*2* mostly expressed in carpels, fruits and leaves, suggesting they are not involved in specifying perianth identity [23].

### E-class genes have broad expression patterns suggestive of the maintenance of their ancestral role in *B. frutescens*

The E class genes *SEPALLATA* (*SEP*) are unique to angiosperms where they have duplicated resulting in the *SEP3* and the *SEP1/2/4* (also named *LOFSEP*) clades [19]. *SEP* genes in *Arabidopsis* have been shown to play redundant roles on meristem and floral organ identity as the quadruple mutant shows a homeotic transformation from floral organs to leaves [14, 60]. *SEP3* orthologs, like *SEP3,* have remained as single copy genes, are central in protein–protein interactions (PPIs) and are evolving under negative selection [61–64]. On the other hand, *LOFSEP* orthologs have duplicated in eudicots and monocots, are more labile in their protein interactions and can evolve under positive selection [64, 65]. *B. frutescens* has two *SEP3* copies (*BofrSEP3*-*1* and *BofrSEP3*-*2*) and three *LOFSEP* copies (*BofrSEP1*-*1*, *BofrSEP1*-*2* and *BofrSEP2*). *BofrSEP3*-*2* is expressed at higher levels than *BofrSEP3*-*1,* and they are mostly restricted to stamens and carpels (Fig. 8j). This suggests that *BofrSEP3*-2 may be the key factor mediating PPIs with *AP3* and *AG* homologs, acting similarly to *SEP3* in *Arabidopsis*, but is likely not involved in sepal identity [66–68]. From the *LOFSEP* paralogs, *BofrSEP1*-*1* is expressed in all floral organs, fruits and leaves, which suggests that this paralog could be performing roles attributed to other *LOFSEP* homologs, including floral meristem and floral organ identity as well as fruit development. Similar expression patterns have been observed in monocots, where *SEP* copies have overlapping, although variable, expression patterns that are suggestive of both function redundancy and subfunctionalization among copies [62, 64, 69].

## Conclusions

Our study integrating developmental /morphological data as well as gene expression and evolution data points to a number of conclusions regarding the natural homeosis occurring in petal-less poppies: (1) Petal loss in poppies may be acquired via distinct developmental pathways with complete homeosis in the second and third whorls (like in *M. cordata*) or complete homeosis in the second floral whorl, accompanied by abortion in the third floral whorl (like in *B. frutescens*). Interestingly, naturally occurring *B. frutescens* natural mutants never show the petal-to-sepal homeosis (predicted by the model), but a petal-to-stamen homeosis, suggesting that other negative regulatory loops between B and C class genes have likely not been identified. (2) The lack of *AP3*-*3* gene expression in the apetalous *B. frutescens* is consistent with the reported role in petal identity of *AP3*-*3* orthologs in Papaveraceae. This has been previously well established for Ranunculaceae and supports the idea that subfunctionalization in the *AP3* paralogs occurred early in the Ranunculales, prior to the diversification of Papaveraceae and Ranunculaceae.

## Additional files

10.1186/s13227-016-0054-6 Accession numbers of all sequences included in the phylogenetic analyses.
10.1186/s13227-016-0054-6 Primer sequences for RT-PCR and qRT-PCR amplification used in this research project.
10.1186/s13227-016-0054-6. SEM micrographs of *Bocconia frutescens* terminal flower. A. Young inflorescence; note the terminal flower larger than the lateral flowers. B. Terminal flower with three sepals. C-E. Initiation of sepals. F. Initiation of the first whorl of homeotic stamens (*_1_). G. Initiation of the first whorl of true stamens alternate to the sepals (1). H-I. Initiation of the second whorl of true stamens (2) belonging to the first whorl, sometimes developed from a common primordium, followed by the third set of true stamens belonging to the second whorl (3). J-K. Initiation of the ovule. L. Late development showing a persistent sepal covering the rest of the floral organs. Bars: A, J, L = 100 µm; B, C, F = 10 µm; D, E, G-I, K = 50 µm.

## Abbreviations

ACT2: ACTIN2
AP3: APETALA3
AG: AGAMOUS
CPD: Critical point dryer
FUL: FRUITFULL
EF1α: ELONGATION FACTOR 1α
GADPH: Glyceraldehyde 3-phosphate dehydrogenase
ML: Maximum likelihood
PI: PISTILLATA
qRT-PCR: Quantitative reverse transcriptase polymerase chain reaction
RT-PCR: Reverse transcriptase polymerase chain reaction
SEM: Scanning electron microcopy
SEP: SEPALLATA
