# Supplementary material for: The developmental and genetic bases of apetaly in Bocconia frutescens (Chelidonieae: Papaveraceae)
Source: EvoDevo. 2016 Aug 2;7:16. doi: 10.1186/s13227-016-0054-6 (PMC4971710; doi:10.1186/s13227-016-0054-6)
Supplement: Supplementary file 1 — 10.1186/s13227-016-0054-6 Accession numbers of all sequences included in the phylogenetic analyses. [file 13227_2016_54_MOESM1_ESM.docx]

**Supplementary Table 1.**

Accession numbers of all sequences included in the phylogenetic analyses

Accession numbers of *FRUITFULL* sequences used in this study.

| *BebeFL1* | *Berberis bealei* | Berberidaceae | JN593334 |
| --- | --- | --- | --- |
| *BgilFL1* | *Berberis gilgiana* | Berberidaceae | KF500145 |
| *BgilFL2* | *Berberis gilgiana* | Berberidaceae | KF500142 |
| *BgilFL3* | *Berberis gilgiana* | Berberidaceae | KF500143 |
| *BgilFL4* | *Berberis gilgiana* | Berberidaceae | KF500144 |
| *BthunFl1* | *Berberis thunbergii* | Berberidaceae | KF500149 |
| *DpFL* | *Dysosma pleiantha* | Berberidaceae | JN593333 |
| *EsagFL1* | *Epimedium sagittatum* | Berberidaceae | JN590216 |
| *JediFL1* | *Jefersonia diphylla* | Berberidaceae | KF500146 |
| *NdomFL1* | *Nandina domestica* | Berberidaceae | KF500147 |
| *NdomFL2* | *Nandina domestica* | Berberidaceae | KF500148 |
| *EuplFL1* | *Euptelea pleiosperma* | Eupteleaceae | DQ656558 |
| *EuplFL2* | *Euptelea pleiosperma* | Eupteleaceae | DQ656559 |
| *AktFL1* | *Akebia trifoliata* | Lardizabalaceae | AY627632 |
| *AktFL2* | *Akebia trifoliata* | Lardizabalaceae | GU357459 |
| *DEinFL1* | *Decaisnea insignis* | Lardizabalaceae | DQ656556 |
| *DEinFL2* | *Decaisnea insignis* | Lardizabalaceae | DQ656557 |
| *SIchFL1* | *Sinofranchetia chinensis* | Lardizabalaceae | DQ656565 |
| *SIchFL2* | *Sinofranchetia chinensis* | Lardizabalaceae | DQ656566 |
| *MencanFL1* | *Menispermum canadense* | Menispermaceae | KF500150 |
| *MencanFL2* | *Menispermum canadense* | Menispermaceae | KF500170 |
| *ArmeFL1* | *Argemone mexicana* | Papaveraceae | KF500126 |
| *ArmeFL2* | *Argemone mexicana* | Papaveraceae | KF500127 |
| *ArmeFL3* | *Argemone mexicana* | Papaveraceae | KF500163 |
| *ArmeFL4* | *Argemone mexicana* | Papaveraceae | KF500162 |
| *BofrFL1* | *Bocconia frutescens* | Papaveraceae | KF500160 / comp 17547 seq 12 |
| *BofrFL2* | *Bocconia frutescens* | Papaveraceae | KF500161 / comp 17547 seq 11 |
| *BofrFL3* | *Bocconia frutescens* | Papaveraceae | comp 15838 |
| *CaseFL1* | *Capnoides sempervirens* | Papaveraceae | KF500118 |
| *CmFL1* | *Chelidonium majus* | Papaveraceae | AY306144 |
| *CmFL2* | *Chelidonium majus* | Papaveraceae | AY306145 |
| *CocheFL1* | *Corydalis cheilanthifolia* | Papaveraceae | KF500119 |
| *CocheFL2* | *Corydalis cheilanthifolia* | Papaveraceae | KF500154 |
| *CyveFL1* | *Cysticapnos vesicaria* | Papaveraceae | KF500121 |
| *DascaFL1* | *Dactylicapnos scandens* | Papaveraceae | KF500120 |
| *DascaFL2* | *Dactylicapnos scandens* | Papaveraceae | KF500151 |
| *DexiFL1* | *Dicentra eximia* | Papaveraceae | KF500115 |
| *DexiFL2* | *Dicentra eximia* | Papaveraceae | KF500155 |
| *EchiFL1* | *Eomecon chionantha* | Papaveraceae | KF500122 |
| *EscaFL1* | *Eschscholzia californica* | Papaveraceae | HM592297 |
| *EscaFL2* | *Eschscholzia californica* | Papaveraceae | HM592298 |
| *EscaFL3* | *Eschscholzia californica* | Papaveraceae | KF500168 |
| *EscaFL4* | *Eschscholzia californica* | Papaveraceae | ECARTPF1 comp 19495 |
| *LaspecFL1* | *Lamprocapnos spectabilis* | Papaveraceae | KF500114 |
| *LaspecFL2* | *Lamprocapnos spectabilis* | Papaveraceae | KF500152 |
| *MacoFL1* | *Macleaya cordata* | Papaveraceae | KF500124 |
| *MacoFL2* | *Macleaya cordata* | Papaveraceae | KF500123 |
| *MacoFL3* | *Macleaya cordata* | Papaveraceae | KF500158 |
| *MacoFL4* | *Macleaya cordata* | Papaveraceae | KF500159 |
| *MecaFL1* | *Meconopsis cambrica* | Papaveraceae | KF500129 |
| *MecaFL2* | *Meconopsis cambrica* | Papaveraceae | KF500165 |
| *PatlaFL1* | *Papaver atlanticum* | Papaveraceae | KF500132 |
| *PbracFL1* | *Papaver bracteatum* | Papaveraceae | KF500131 |
| *PbracFL2* | *Papaver bracteatum* | Papaveraceae | KF500166 |
| *PapnFL1* | *Papaver nudicaule* | Papaveraceae | AY306175 |
| *PrhFL1* | *Papaver rhoeas* | Papaveraceae | KF500130 |
| *PrhFL2* | *Papaver rhoeas* | Papaveraceae | KF500167 |
| *PapsFL1* | *Papaver somniferum* | Papaveraceae | AY306177 |
| *PapsFL2* | *Papaver somniferum* | Papaveraceae | AY306178 |
| *ClutFL1* | *Pseudofumaria lutea* | Papaveraceae | KF500117 |
| *ClutFL2* | *Pseudofumaria lutea* | Papaveraceae | KF500116 |
| *ClutFL3* | *Pseudofumaria lutea* | Papaveraceae | KF500153 |
| *RocoFL1* | *Romneya coulteri* | Papaveraceae | KF500128 |
| *RocoFL2* | *Romneya coulteri* | Papaveraceae | KF500164 |
| *ScanFL2* | *Sanguinaria canadensis* | Papaveraceae | KF500157 |
| *SdyFL1* | *Stylophorum diphyllum* | Papaveraceae | KF500125 |
| *SdyFL2* | *Stylophorum diphyllum* | Papaveraceae | KF500156 |
| *AnsylFL1* | *Anemone sylvestris* | Ranunculaceae | KF500141 |
| *AqFLA* | *Aquilegia coerulea* | Ranunculaceae | KF500133 |
| *AqFLB* | *Aquilegia coerulea* | Ranunculaceae | KF500134 |
| *ClejoeFL1* | *Clematis marmoraria x clematis paniculata cultivar Joe* | Ranunculaceae | KF500139 |
| *ErhyeFL1* | *Eranthis hyemalis* | Ranunculaceae | KF500138 |
| *ErhyeFL2* | *Eranthis hyemalis* | Ranunculaceae | KF500140 |
| *HehyFL1* | *Helleborus x hibridus* | Ranunculaceae | KF500135 |
| *HehyFL2* | *Helleborus x hibridus* | Ranunculaceae | KF500171 |
| *HycaFL1* | *Hydrastis canadensis* | Ranunculaceae | KF500136 |
| *HycaFl2* | *Hydrastis canadensis* | Ranunculaceae | KF500172 |
| *NisaFL1* | *Nigella sativa* | Ranunculaceae | KF500137 |
| *NisaFL2* | *Nigella sativa* | Ranunculaceae | KF500169 |
| *RbFL1* | *Ranunculus bulbosus* | Ranunculaceae | AY306179 |
| *RbFL2* | *Ranunculus bulbosus* | Ranunculaceae | AY306180 |
| *RbFL3* | *Ranunculus bulbosus* | Ranunculaceae | AY306182 |
| *RbFL4* | *Ranunculus bulbosus* | Ranunculaceae | AY306183 |
| *RascFL1* | *Ranunculus sceleratus* | Ranunculaceae | AB473875 |
| *RascFL2* | *Ranunculus sceleratus* | Ranunculaceae | AB473876 |
| *RascFL3* | *Ranunculus sceleratus* | Ranunculaceae | AB473877 |

Accession numbers of *APETALA3* sequences used in this study.

| *BgAP3-1* | *Berberis gilgiana* | Berberidaceae | AY162857 |
| --- | --- | --- | --- |
| *BgAP3-2* | *Berberis gilgiana* | Berberidaceae | AY162958 |
| *EpgAP3-1* | *Epimedium grandiflora* | Berberidaceae | EU481793 |
| *EpgAP3-3* | *Epimedium grandiflora* | Berberidaceae | EU481794 |
| *JdAP3-1* | *Jeffersonia diphylla* | Berberidaceae | EU481791 |
| *CiaAP3a* | *Circaeaster agrestis* | Circaeasteraceae | HQ647369 |
| *CiaAP3b* | *Circaeaster agrestis* | Circaeasteraceae | HQ647370 |
| *KiuAP3a* | *Kingdonia uniflora* | Circaeasteraceae | HQ647371 |
| *KiuAP3b* | *Kingdonia uniflora* | Circaeasteraceae | HQ647372 |
| *EupAP3* | *Euptelea polyandra* | Eupteleaceae | EU481781 |
| *EupAP3-1* | *Euptelea pleiosperma* | Eupteleaceae | HQ647367 |
| *EupAP3-2* | *Euptelea pleiosperma* | Eupteleaceae | HQ647368 |
| *AkqAP3-1* | *Akebia quinata* | Lardizabalaceae | AY162835 |
| *AkqAP3-2* | *Akebia quinata* | Lardizabalaceae | AY162839 |
| *HbcAP3-1* | *Holboellia coriacea* | Lardizabalaceae | EU481789 |
| *HbcAP3-2* | *Holboellia coriacea* | Lardizabalaceae | EU481788 |
| *CctAP3-1* | *Cocculus trilobus* | Menispermaceae | HQ694788 |
| *CctAP3-2* | *Cocculus trilobus* | Menispermaceae | HQ694789 |
| *CctAP3-3* | *Cocculus trilobus* | Menispermaceae | HQ694790 |
| *MndAP3-1* | *Menispermum dauricum* | Menispermaceae | EU481786 |
| *MndAP3-2* | *Menispermum dauricum* | Menispermaceae | EU481784 |
| *MndAP3-3* | *Menispermum dauricum* | Menispermaceae | EU481783 |
| *ArmeAP3-1* | *Argemone Mexicana* | Papaveraceae | IRAF 2012353 |
| *ArmeAP3-3* | *Argemone Mexicana* | Papaveraceae | IRAF 2000627 |
| *BofrAP3* | *Bocconia frutescens* | Papaveraceae | Comp 7143 |
| *CaseAP3-1* | *Capnoides sempervirens* | Papaveraceae | AUGU 2007225 |
| *CeveAP3-1* | *Ceratocapnos vesicaria* | Papaveraceae | UDHA 2015403 |
| *CeveAP3-3* | *Ceratocapnos vesicaria* | Papaveraceae | UDHA 2096224 |
| *DeAP3* | *Dicentra eximia* | Papaveraceae | AF052875 |
| *EcDEF1* | *Eschscholzia californica* | Papaveraceae | EF378697 |
| *EcDEF2* | *Eschscholzia californica* | Papaveraceae | EF378698 |
| *EcDEF3* | *Eschscholzia californica* | Papaveraceae | HE573239 |
| *PnAP3-1* | *Papaver nudicaule* | Papaveraceae | AF052873 |
| *PnAP3-2* | *Papaver nudicaule* | Papaveraceae | AF052874 |
| *PrhoAP3-1* | *Papaver rhoeas* | Papaveraceae | IORZ 2022756 |
| *PrhoAP3-3* | *Papaver rhoeas* | Papaveraceae | IORZ 2125649 |
| *PaseAP3-1* | *Papaver setigerum* | Papaveraceae | JSVC 2036598 |
| *PaseAP3-3* | *Papaver setigerum* | Papaveraceae | QCOU 2076458 |
| *PapsAP3-1* | *Papaver somniferum* | Papaveraceae | EF071993 |
| *PapsAP3-2* | *Papaver somniferum* | Papaveraceae | EF071992 |
| *ScAP3* | *Sanguinaria canadensis* | Papaveraceae | AF130868 |
| *ScAP3-2* | *Sanguinaria canadensis* | Papaveraceae | XHKT 2004515 |
| *ScAP3-3* | *Sanguinaria canadensis* | Papaveraceae | XHKT 2005759 |
| *Sdy AP3-1* | *Stylophorum diphyllum* | Papaveraceae | 22739930 comp 40800 |
| *SdyAP3-2* | *Stylophorum diphyllum* | Papaveraceae | 22743916 comp 11396 |
| *AcsAP3-1* | *Aconitum sinomontanum* | Ranunculaceae | EU481818 |
| *AcsAP3-2* | *Aconitum sinomontanum* | Ranunculaceae | EU481817 |
| *AcsAP3-3* | *Aconitum sinomontanum* | Ranunculaceae | EU481816 |
| *AcaAP3-1* | *Actaea asiatica* | Ranunculaceae | HQ647375 |
| *AcaAP3-2* | *Actaea asiatica* | Ranunculaceae | HQ647376 |
| *AdvAP3-2* | *Adonis vernalis* | Ranunculaceae | HQ694800 |
| *AdvAP3-3* | *Adonis vernalis* | Ranunculaceae | HQ694799 |
| *AnnAP3-1* | *Anemone nemerosa* | Ranunculaceae | AY162841 |
| *AnnAP3-2* | *Anemone nemerosa* | Ranunculaceae | AY162842 |
| *AnnAP3-3* | *Anemone nemerosa* | Ranunculaceae | AY162843 |
| *AqAP3-3b* | *Aquilegia coerulea* | Ranunculaceae | HQ694798 |
| *AqvAP3-1* | *Aquilegia vulgaris* | Ranunculaceae | EF489478 |
| *AqvAP3-2* | *Aquilegia vulgaris* | Ranunculaceae | EF489477 |
| *AqvAP3-3* | *Aquilegia vulgaris* | Ranunculaceae | EF489476 |
| *CapAP3-1* | *Caltha palustris* | Ranunculaceae | EU481813 |
| *CapAP3-2* | *Caltha palustris* | Ranunculaceae | EU481812 |
| *CirAP3-1* | *Cimicifuga racemosa* | Ranunculaceae | AY162862 |
| *CirAP3-2* | *Cimicifuga racemosa* | Ranunculaceae | AY162863 |
| *CirAP3-3* | *Cimicifuga racemosa* | Ranunculaceae | AY162864 |
| *ClaAP3-1* | *Clematis alpina* | Ranunculaceae | EU481809 |
| *ClaAP3-2* | *Clematis alpina* | Ranunculaceae | EU481807 |
| *ClaAP3-3* | *Clematis alpina* | Ranunculaceae | EU481806 |
| *CliAP3-1* | *Clematis integrifolia* | Ranunculaceae | AY162870 |
| *CliAP3-2* | *Clematis integrifolia* | Ranunculaceae | AY162871 |
| *DleAP3-1* | *Delphinium exaltatum* | Ranunculaceae | EU481804 |
| *DleAP3-2* | *Delphinium exaltatum* | Ranunculaceae | EU481803 |
| *DleAP3-3* | *Delphinium exaltatum* | Ranunculaceae | EU481802 |
| *HoAP3-1* | *Helleborus orientalis* | Ranunculaceae | AY162874 |
| *HoAP3-2* | *Helleborus orientalis* | Ranunculaceae | AY162875 |
| *HoAP3-3a* | *Helleborus orientalis* | Ranunculaceae | AY162876 |
| *HoAP3-3b* | *Helleborus orientalis* | Ranunculaceae | AY162877 |
| *HehAP3-1* | *Hepatica henryi* | Ranunculaceae | HQ647378 |
| *HehAP3-2* | *Hepatica henryi* | Ranunculaceae | HQ647379 |
| *HycAP3-1* | *Hydrastis Canadensis* | Ranunculaceae | EU481800 |
| *NgsAP3-2* | *Nigella sativa* | Ranunculaceae | HQ694795 |
| *NgsAP3-3* | *Nigella sativa* | Ranunculaceae | HQ694794 |
| *RfAP3-1* | *Ranunculus ficaria* | Ranunculaceae | AF052854 |
| *RfAP3-2* | *Ranunculus ficaria* | Ranunculaceae | AF130870 |
| *RfAP3-3* | *Ranunculus ficaria* | Ranunculaceae | AY162883 |
| *RbAP3-1* | *Ranunculus bulbosus* | Ranunculaceae | AF052876 |
| *RbAP3-2* | *Ranunculus bulbosus* | Ranunculaceae | AF130869 |
| *ThdAP3-1* | *Thalictrum dioicum* | Ranunculaceae | AY867875 |
| *ThdAP3-2a* | *Thalictrum dioicum* | Ranunculaceae | AY867876 |
| *ThdAP3-2b* | *Thalictrum dioicum* | Ranunculaceae | AY867877 |
| *ThtAP3-1* | *Thalictrum thalictroides* | Ranunculaceae | AY162886 |
| *ThtAP3-2a* | *Thalictrum thalictroides* | Ranunculaceae | AY162887 |
| *ThtAP3-2b* | *Thalictrum thalictroides* | Ranunculaceae | AY162888 |
| *TrcAP3* | *Trautvetteria carolinensis* | Ranunculaceae | AY162904 |
| *TllAP3-1* | *Trollius laxus* | Ranunculaceae | AY162890 |
| *TllAP3-2* | *Trollius laxus* | Ranunculaceae | AY162892 |
| *TllAP3-3* | *Trollius laxus* | Ranunculaceae | AY162894 |
| *XsAP3-2* | *Xanthoriza simplicissima* | Ranunculaceae | EU481797 |
| *XsAP3-3* | *Xanthoriza simplicissima* | Ranunculaceae | EU481796 |

Accession numbers of *PISTILLATA* sequences used in this study.

| *BgPI-1* | *Berberis gilgiana* | Berberidaceae | AY162860 |
| --- | --- | --- | --- |
| *BgPI-2* | *Berberis gilgiana* | Berberidaceae | AY162861 |
| *EpgPI* | *Epimedium grandiflora* | Berberidaceae | EU481795 |
| *JdPI* | *Jeffersonia diphylla* | Berberidaceae | EU481792 |
| *NndPI* | *Nandina domestica* | Berberidaceae | HQ694793 |
| *PdcPI* | *Podophyllum canadensis* | Berberidaceae | HQ694792 |
| *CiaPI* | *Circaeaster agrestis* | Circaeasteraceae | HQ647374 |
| *KiuPI* | *Kingdonia uniflora* | Circaeasteraceae | HQ647373 |
| *EupPI* | *Euptelea polyandra* | Eupteleaceae | EU481782 |
| *AkqPI* | *Akebia quinata* | Lardizabalaceae | AY162837 |
| *HbcPI* | *Holboellia coriacea* | Lardizabalaceae | EU481790 |
| *CctPI* | *Cocculus trilobus* | Menispermaceae | HQ694791 |
| *MndPI* | *Menispermum dauricum* | Menispermaceae | EU481787 |
| *ArmePI-1* | *Argemone Mexicana* | Papaveraceae | IRAF 2021442 |
| *ArmePI-2* | *Argemone Mexicana* | Papaveraceae | IRAF 2000626 |
| *BofrPI1* | *Bocconia frutescens* | Papaveraceae | Comp 16377 seq 2 |
| *BofrPI2* | *Bocconia frutescens* | Papaveraceae | Comp 16377 seq 6 |
| *BofrPI3* | *Bocconia frutescens* | Papaveraceae | Comp 16377 seq 4 |
| *BofrPI4* | *Bocconia frutescens* | Papaveraceae | Comp 16377 seq 3 |
| *DePI* | *Dicentra eximia* | Papaveraceae | AF052857 |
| *CasePI* | *Capnoides sempervirens* | Papaveraceae | AUGV 2007225 |
| *CasePI2* | *Capnoides sempervirens* | Papaveraceae | AUGV 2049607 |
| *CevePI-1* | *Ceratocapnos vesicaria* | Papaveraceae | UDMA 2013443 |
| *CevePI-3* | *Ceratocapnos vesicaria* | Papaveraceae | UDMA 2013442 |
| *EcGLO* | *Eschscholzia californica* | Papaveraceae | EF378699 |
| *HyproPI-1* | *Hypecoum procumbens* | Papaveraceae | NMGG 2013387 |
| *HyproPI-2* | *Hypecoum procumbens* | Papaveraceae | NMGG 2013386 |
| *PbraPI* | *Papaver bracteatum* | Papaveraceae | TMWU 2021350 |
| *PnPI-1* | *Papaver nudicaule* | Papaveraceae | AF052855 |
| *PnPI-2* | *Papaver nudicaule* | Papaveraceae | AF052856 |
| *PrhoPI-1* | *Papaver rhoeas* | Papaveraceae | MVTX 2016817 |
| *PrhoPI-2* | *Papaver rhoeas* | Papaveraceae | IORZ 2125649 |
| *PasePI-1* | *Papaver setigerum* | Papaveraceae | JSVC 2022464 |
| *PasePI-2* | *Papaver setigerum* | Papaveraceae | QCOU 2076458 |
| *PapsPI-1* | *Papaver somniferum* | Papaveraceae | EF071994 |
| *PapsPI-2* | *Papaver somniferum* | Papaveraceae | EF071995 |
| *ScPI* | *Sanguinaria canadensis* | Papaveraceae | AF130871 |
| *ScPI-2* | *Sanguinaria canadensis* | Papaveraceae | XHKT 2009840 |
| *AcsPI* | *Aconitum sinomontanum* | Ranunculaceae | EU481820 |
| *AcaPI* | *Actaea asiatica* | Ranunculaceae | HQ647377 |
| *AdvPI-1* | *Adonis vernalis* | Ranunculaceae | HQ694801 |
| *AdvPI-2* | *Adonis vernalis* | Ranunculaceae | HQ694802 |
| *AnnPI-1* | *Anemone nemerosa* | Ranunculaceae | AY162845 |
| *AnnPI-2* | *Anemone nemerosa* | Ranunculaceae | AY162847 |
| *AqvPI* | *Aquilegia vulgaris* | Ranunculaceae | EF489475 |
| *CapPI* | *Caltha palustris* | Ranunculaceae | EU481815 |
| *CirPI-1* | *Cimicifuga racemosa* | Ranunculaceae | AY162865 |
| *CirPI-2* | *Cimicifuga racemosa* | Ranunculaceae | AY162867 |
| *ClaPI-1* | *Clematis alpina* | Ranunculaceae | EU481811 |
| *ClaPI-2* | *Clematis alpina* | Ranunculaceae | EU481810 |
| *CliPI-1* | *Clematis integrifolia* | Ranunculaceae | AY162872 |
| *CliPI-2* | *Clematis integrifolia* | Ranunculaceae | AY162873 |
| *DlePI* | *Delphinium exaltatum* | Ranunculaceae | EU481805 |
| *HoPI-1* | *Helleborus orientalis* | Ranunculaceae | AY162878 |
| *HoPI-2* | *Helleborus orientalis* | Ranunculaceae | AY162879 |
| *HoPI-3* | *Helleborus orientalis* | Ranunculaceae | AY162880 |
| *HehPI* | *Hepatica henryi* | Ranunculaceae | HQ647380 |
| *HycPI* | *Hydrastis Canadensis* | Ranunculaceae | EU481801 |
| *NgsPI* | *Nigella sativa* | Ranunculaceae | HQ694797 |
| *NgsPI* | *Nigella sativa* | Ranunculaceae | HQ694796 |
| *RfPI-1* | *Ranunculus ficaria* | Ranunculaceae | AF052858 |
| *RfPI-1b* | *Ranunculus ficaria* | Ranunculaceae | AY162884 |
| *RfPI-2* | *Ranunculus ficaria* | Ranunculaceae | AF130872 |
| *RfPI-3* | *Ranunculus ficaria* | Ranunculaceae | AY162885 |
| *RbPI-1* | *Ranunculus bulbosus* | Ranunculaceae | AF052859 |
| *RbPI-2* | *Ranunculus bulbosus* | Ranunculaceae | AF052860 |
| *ThtPI* | *Thalictrum thalictroides* | Ranunculaceae | AY162889 |
| *TrcPI-1* | *Trautvetteria carolinensis* | Ranunculaceae | AY162905 |
| *TrcPI-2* | *Trautvetteria carolinensis* | Ranunculaceae | AY162906 |
| *TllPI-1* | *Trollius laxus* | Ranunculaceae | AY162896 |
| *TllPI-2* | *Trollius laxus* | Ranunculaceae | AY162898 |
| *TllPI-3* | *Trollius laxus* | Ranunculaceae | AY162901 |
| *TllPI-4* | *Trollius laxus* | Ranunculaceae | AY162902 |
| *XsPI-1* | *Xanthoriza simplicissima* | Ranunculaceae | EU481799 |
| *XsPI-2* | *Xanthoriza simplicissima* | Ranunculaceae | EU481798 |

Accession numbers of *AGAMOUS/SEEDTICK* sequences used in this study.

| *BgilAG* | *Berberis gilgiana* | Berberidaceae | AY464106 |
| --- | --- | --- | --- |
| *NadoAG* | *Nandina domestica* | Berberidaceae | YHFG 2075401 |
| *EuplAG1* | *Euptelea pleiosperma* | Eupteleaceae | GU357452 |
| *EuplAG2* | *Euptelea pleiosperma* | Eupteleaceae | GU357453 |
| *AkquAG* | *Akebia quinata* | Lardizabalaceae | AY464107 |
| *AktrAG* | *Akebia trifoliata* | Lardizabalaceae | AY627635 |
| *HogrAG1* | *Holboellia grandiflora* | Lardizabalaceae | JQ806406 |
| *HogrAG2* | *Holboellia grandiflora* | Lardizabalaceae | JQ806407 |
| *ArmeAG1* | *Argenome mexicana* | Papaveraceae | BFMT 2006242 |
| *ArmeAG2* | *Argenome mexicana* | Papaveraceae | BFMT 2006241 |
| *ArmeAG3* | *Argenome mexicana* | Papaveraceae | BMFT 2012280 |
| *BofrAG1* | *Bocconia frutescens* | Papaveraceae | comp 14958 seq 1 |
| *EscaAG1* | *Eschscholzia californica* | Papaveraceae | DQ088996 |
| *EscaAG2* | *Eschscholzia californica* | Papaveraceae | DQ088997 |
| *PapsAG1* | *Papaver somniferum* | Papaveraceae | GU123602 |
| *PapsAG2* | *Papaver somniferum* | Papaveraceae | GU123603 |
| *PaseAG1* | *Papaver setigerum* | Papaveraceae | EPRK 2005601 |
| *PaseAG2* | *Papaver setigerum* | Papaveraceae | EPRK 2005600 |
| *PrhoAG1* | *Papaver rhoeas* | Papaveraceae | BEKN 2018230 |
| *PrhoAG2* | *Papaver rhoeas* | Papaveraceae | BEKN 2018229 |
| ***ScAG*** | *Sanguinaria canadensis* | Papaveraceae | AY464097 |
| *ScAG2* | *Sanguinaria canadensis* | Papaveraceae | XHKT-2007933 |
| *AnpuAG* | *Anemone pulsatila* | Ranunculaceae | UPOG 2047206 |
| *AqaAG1* | *Aquilegia alpina* | Ranunculaceae | AY464110 |
| *AqaAG2* | *Aquilegia alpina* | Ranunculaceae | AY464111 |
| *AqcAG1** | *Aquilegia coerulea* | Ranunculaceae | Aquca-022-00039.1 |
| *AqcAG2** | *Aquilegia coerulea* | Ranunculaceae | Aquca-136-00009.1 |
| *CliAG1* | *Clematis integrifolia* | Ranunculaceae | AY464113 |
| *CliAG2* | *Clematis integrifolia* | Ranunculaceae | AY464112 |
| *HycaAG1* | *Hydrastis canadensis* | Ranunculaceae | HCARTLPF comp 7044 |
| *RfAG1* | *Ranunculus ficaria* | Ranunculaceae | AY464115 |
| *RfAG2* | *Ranunculus ficaria* | Ranunculaceae | AY464114 |
| *ThdAG1* | *Thalictrum dioicum* | Ranunculaceae | AY464095 |
| *ThdAG1a* | *Thalictrum dioicum* | Ranunculaceae | AY867872 |
| *ThdAG2* | *Thalictrum dioicum* | Ranunculaceae | AY464094 |
| *ThtAG1* | *Thalictrum thalictroides* | Ranunculaceae | JN887118 |
| *ThtAG2* | *Thalictrum thalictroides* | Ranunculaceae | AY867879 |

| *AktrSTK* | *Akebia trifoliata* | Lardizabalaceae | AY627629 |
| --- | --- | --- | --- |
| *CeveSTK* | *Ceratocapnos vesicaria* | Papaveraceae | UDHA 2093761 |
| *EscaAGL11* | *Eschscholzia californica* | Papaveraceae | DQ088998 |
| *HyproSTK* | *Hypecoum procumbens* | Papaveraceae | NMGG 2061825 |
| *PrhoSTK* | *Papaver rhoeas* | Papaveraceae | IORZ 2125324 |
| *PaseSTK* | *Papaver setigerum* | Papaveraceae | STDO 2040306 |
| *PapsSTK* | *Papaver somniferum* | Papaveraceae | RQNK 2021003 |
| *ScanSTK* | *Sanguinaria canadensis* | Papaveraceae | XHKT 2009840 |

* These sequences of AGAMOUS isolated from *Aquilegia coerulea* (*Aqc*) were labelled differently in a previous analysis. *AqcAG2*, was labelled as *AqAG1* and *AqcAG1* was labelled as *AqAG2* (see Pabón Mora et al 2014, Figure 5 and supplementary data). Unlike this analysis the previous one was not aimed at identifying local duplications and therefore had a limted sampling in Ranunculaceae. Here are the sequences with the correct naming and their identifier in databases.

Accession numbers of *SEPALLATA* sequences used in this study.

| *BejuSEP3* | *Berberis julianae* | Berberidaceae | HM121964 |
| --- | --- | --- | --- |
| *NadoSEP3* | *Nandina domestica* | Berberidaceae | YHFG 2002253 |
| *EsAGL2-1* | *Epimedium sagittatum* | Berberidaceae | JN590218 |
| *EsAGL2-2* | *Epimedium sagittatum* | Berberidaceae | JN590219 |
| *EuplSEP1* | *Euptelea pleiosperna* | Eupteleaceae | GU357461 |
| *EuplSEP3* | *Euptelea pleiosperna* | Eupteleaceae | GU357460 |
| *AktrSEP1* | *Akebia trifoliata* | Lardizabalaceae | GU357447 |
| *AktrSEP1-2* | *Akebia trifoliata* | Lardizabalaceae | GU357448 |
| *AktrSEP3-1* | *Akebia trifoliata* | Lardizabalaceae | AY627628 |
| *SichSEP3* | *Sinofranchetia chinensis* | Lardizabalaceae | JQ806399 |
| *ColoSEP1* | *Cocculus laurifolluss* | Menispermaceae | LVNW 2017507 |
| *ColaSEP3* | *Cocculus laurifolluss* | Menispermaceae | LVNW 2015542 |
| *MecaSEP1* | *Menispermun canadense* | Menispermaceae | MCARHIPH comp 13188 |
| *MecaSEP2* | *Menispermun canadense* | Menispermaceae | MCARHIPH comp 44939 |
| *ArmeSEP1* | *Argemone mexicana* | Papaveraceae | CCHG 2016464 |
| *ArmeSEP3* | *Argemone mexicana* | Papaveraceae | CCHG 2016463 |
| *ArmeSEP32* | *Argemone mexicana* | Papaveraceae | BFMT 2013064 |
| *BofrSEP3-1* | *Bocconia frutescens* | Papaveraceae | Comp 14408 seq 1 |
| *BofrSEP3-2* | *Bocconia frutescens* | Papaveraceae | Comp 14408 seq 2 |
| *BofrSEP3* | *Bocconia frutescens* | Papaveraceae | Comp 15699 seq 1 |
| *BofrSEP1-1* | *Bocconia frutescens* | Papaveraceae | Comp 19151 seq 24 |
| *BofrSEP1-2* | *Bocconia frutescens* | Papaveraceae | Comp 19151 seq 19 |
| *CmaSEP1* | *Chelidonium majus* | Papaveraceae | XMVD 2046470 |
| *CmaSEP3* | *Chelidonium majus* | Papaveraceae | CMAST2PF comp 9428 |
| *CaseSEP3* | *Capnoides sempervirens* | Papaveraceae | AUGV 2000569 |
| *CeveSEP1* | *Ceratocapnos vesicaria* | Papaveraceae | UDHA 2016816 |
| *EscaAGL2* | *Eschoscholzia californica* | Papaveraceae | AY850181/ERXG 2063182 |
| *EscaAGL9* | *Eschoscholzia californica* | Papaveraceae | AY850180/ERXG 2015707 |
| *HyproSEP1* | *Hypecoum procumbens* | Papaveraceae | NMGG 2006350 |
| *HyproSEP3* | *Hypecoum procumbens* | Papaveraceae | NMGG 2010983 |
| *PbracSEP1* | *Papaver bracteatum* | Papaveraceae | SSDU 2028501 |
| *PbracSEP2* | *Papaver bracteatum* | Papaveraceae | SSDU 2146447 |
| *PbracSEP3* | *Papaver bracteatum* | Papaveraceae | SSDU 2161299 |
| *PrhoSEP3* | *Papaver rhoeas* | Papaveraceae | ACYX 2127855 |
| *PrhoSEP32* | *Papaver rhoeas* | Papaveraceae | MTVX 2013876 |
| *PrhoSEP33* | *Papaver rhoeas* | Papaveraceae | QZBA 2013119 |
| *PaseSEP2* | *Papaver setigerum* | Papaveraceae | MLPX 2004140 |
| *PaseSEP3* | *Papaver setigerum* | Papaveraceae | MLPX 2001839 |
| *PapsSEP1* | *Papaver somniferum* | Papaveraceae | SUFP 2007713 |
| *PapsSEP2* | *Papaver somniferum* | Papaveraceae | RQNK 2029979 |
| *PapsSEP3* | *Papaver somniferum* | Papaveraceae | MIKW 2026957 |
| *PapsSEP32* | *Papaver somniferum* | Papaveraceae | KKCW 2018807 |
| *ScanSEP1* | *Sanguinaria canadensis* | Papaveraceae | XHKT 2062447 |
| *ScanSEP2* | *Sanguinaria canadensis* | Papaveraceae | XHKT 2011698 |
| *ScanSEP3* | *Sanguinaria canadensis* | Papaveraceae | XHKT 2063995 |
| *SdiSEP3* | *Stylophorum diphyllum* | Papaveraceae | SDISTIPF comp 24591 |
| *AnneSEP3* | *Anemone nemerosa* | Ranunculaceae | HM121965 |
| *AqcSEP1* | *Aquilegia coerulea* | Ranunculaceae | JX680244 |
| *AqcSEP2A* | *Aquilegia coerulea* | Ranunculaceae | JX680245 |
| *AqcSEP2B* | *Aquilegia coerulea* | Ranunculaceae | JX680246 |
| *CiraSEP1* | *Cimicifuga racemosa* | Ranunculaceae | CYVA 2008475 |
| *CiraSEP12* | *Cimicifuga racemosa* | Ranunculaceae | CYVA 2018839 |
| *CiraSEP3* | *Cimicifuga racemosa* | Ranunculaceae | CYVA 2015924 |
| *HycaSEP3* | *Hydrastis canadensis* | Ranunculaceae | VGHH 2000583 |
| *NisaSEP1* | *Nigella sativa* | Ranunculaceae | NSARTLPF comp 13788 |
| *NisaSEP3* | *Nigella sativa* | Ranunculaceae | NSARTLPF comp 7414 |
| *ThthSEP1* | *Thalictrum thalictroides* | Ranunculaceae | GBVZ 2012287 |
| *ThthSEP2* | *Thalictrum thalictroides* | Ranunculaceae | GBVZ 2006379 |
| *ThthSEP3* | *Thalictrum thalictroides* | Ranunculaceae | GBVZ 2077168 |
