# Supplementary material for: The developmental and genetic bases of apetaly in Bocconia frutescens (Chelidonieae: Papaveraceae)
Source: EvoDevo. 2016 Aug 2;7:16. doi: 10.1186/s13227-016-0054-6 (PMC4971710; doi:10.1186/s13227-016-0054-6)
Supplement: Supplementary file 2 — 10.1186/s13227-016-0054-6 Primer sequences for RT-PCR and qRT-PCR amplification used in this research project. [file 13227_2016_54_MOESM2_ESM.docx]

**Supplementary Table 2**

Primer sequences for RT-PCR and qRT-PCR amplification used in this research project

| **Gene name** | **Primer sequence** | **Target sequence**  **(AA)** | **Amplicon size** | **Tm (C)** |
| --- | --- | --- | --- | --- |
| **RTPCR primers** | | | | |
| *BofrFL1* Fwd | TCGGAATCGCAGGAAATC | SESQXNX | 378 bp | 53.0 |
| *BofrFL1* Rev | GTACCAGTACGAAGTGTTAGC | SLTLRTGT |  | 45.7 |
| *BofrFL2* Fwd | CAGAAATCACAGGGAAGTT | QKSQGS | 471 bp | 45.8 |
| *BofrFL2* Rev | CTATTGGTTGACATGGCCA | LGHVNQ |  | 51.2 |
| *BofrFL3* Fwd | TACTCTTACGCCGAACGGGT | YSYAERV |  | 55.9 |
| *BofrFL3* Rev | TAGCTGGTCCCACTGAATCTG |  |  | 53.9 |
| *BofrAP3*Fwd | CATAATCTCCTACGTGAATTT | HNLLREF | 150 bp | 45.3 |
| *BofrAP3*Rev | TGAAGATTGGGCTGGCTTGG | PSQPNLM |  | 59.5 |
| *Bofr PI1/2/3/4* Fwd | TGATAGTGTTCGCGCCAAAC | YDSVRAKQ | 318bp | 55.9 |
| *BofPI2-PI4* Rev | TTAGGGACAGATGGTGGTGG | TTTICP |  | 54.3 |
| *Bofr* PI1-PI3 Fwd | ACTGTCACCAACAACAAATAG | NCHQQQIE | 205bp | 46.3 |
| *BofrPI1-PI3* Rev | CTATTTGTTGTTGGTGGTAT | NTTNNK |  | 43.8 |
| *Bofr*PI3 Fwd | CCTCACTTACAACTGTCAC | RLTYNCH | 204 bp | 41.1 |
| *Bofr*PI3 Rev | GAACGCAAATGGCATTTGATG | HQMPFAF |  | 57.5 |
| *Bofr*PI2-PI4 Fwd | TTTGTTCACTGCAGCAACAAC | ICSLQQQQ | 225bp | 53.5 |
| *Bofr*PI2-PI4 Rev | TTAGGGACAGATGGTGGTGG | TTTICP |  | 54.3 |
| *Bofr*AG Fwd | ACGAGTACGCTAACAACAGTGT | YEYANNSV | 414 bp | 50.7 |
| *Bofr*AG Rev | AAGTCATGATCGAATTCAT | EFDVMT |  | 43.2 |
| *Bofr*SEP3-1 Fwd | GAAAGGCAGCTTGACATGTCA | ERQLDMS | 301bp | 55.0 |
| *Bofr*SEP3-1 Rev | CCATCCTGGCATGTAACT | SYMPGW |  | 47.2 |
| *Bofr* SEP3-2 Fwd | GAAAGGCAGCTTGACATGTCA | ERQLDMS | 255 bp | 55.0 |
| *Bofr* SEP3-2 Rev | CGTGGGTTCACATTCTATTGG | PIECEPT |  | 54.1 |
| *Bofr*SEP2 Fwd | GAGCAACAATTAGAGTCGTCT | EQQLESS | 318 bp | 47.6 |
| *Bofr*SEP2 Rev | GACGTTTTGGGATGAGTTTGC | ANSSQNV |  | 55.7 |
| *Bofr*SEP1-1 Fwd | GACGTCCAGGATGAAACCTAC | DVQDETY | 335bp | 52.3 |
| *Bofr*SEP1-1 Rev | AGCATTCTGGTGGTTAATATGATA | ISYYHQNA |  | 49.9 |
| *Bofr*SEP1-2 Fwd | GACGTCCAGGATGAAACCTAC | DVQDETY | 277 bp | 52.3 |
| *Bofr*SEP1-2 Rev | AGCATTCTGGTGGTAATATGATA | KVSTLTNV |  | 51.4 |
| *Bofr*ACT Fwd | GATGGATCCTCCAATCCAGACACTGTA |  |  | 62.1 |
| *Bofr*ACT Rev | GTATTGTGTTGGACTCTGGTGATGGTGT |  |  | 62.0 |
| **qRTPCR primers** | | | | |
| Bofr qRT AG Fwd | CAGCACATGAACTTGATGCC |  | 67-150 bp | 58.0 |
| Bofr qRT AG Rev | GCTCTTGGCAGGAGTAATGG |  |  | 55.4 |
| Bofr qRT AP3 Fwd | CACTGAAGGTTGCTCGTGAA |  |  | 54.3 |
| Bofr qRT AP3 Rev | AATCCCCTTCTTGATTGGCT |  |  | 56.0 |
| Bofr qRT PI1-3 Fwd | ATAGTGCAGAATGGGGGTTG |  |  | 58.2 |
| Bofr qRT PI1-3 Rev | GATTGGGTTGAATTGGTTGC |  |  | 55.0 |
| Bofr qRT PI2-4 Fwd | AGGTGGTGGTGGGATTGATA |  |  | 60.0 |
| Bofr qRT PI2-4 Rev | ATGGCATTTGATGATGACGA |  |  | 58.2 |
| Bofr qRT FUL1 Fwd | atttggagcagcagcttgat |  |  | 56.0 |
| Bofr qRT FUL1 Rev | tctttcttcgcctcctacca |  |  | 54.4 |
| Bofr qRT FUL2 Fwd | gcacttccttcttcgcaaac |  |  | 55.0 |
| Bofr qRT FUL2 Rev | atccatggtggcataatcgt |  |  | 56.5 |
| Bofr qRT FUL3 Fwd | GCACTTCCTTCTTCGCAAAC |  |  | 55.0 |
| Bofr qRT FUL3 Rev | TCCAACGTGGCATAATCGTA |  |  | 57.0 |
| ACT Fwd qRT | AAGAGCTCGAAACTGCCAAG |  |  | 58.0 |
| ACT Rev qRT | CATCGGGAAGCTCGTAATTT |  |  | 55.0 |
| GAPHD Fwd qRT | GCTTCCTTCAACATCATTCC |  |  | 56.3 |
| GAPHD Rev qRT | AGTTGCCTTCTTCTCAAGTC |  |  | 55.0 |
| eEF qRT Fwd | AGTCAACTACCACTGGTCAC |  |  | 57.2 |
| eEF qRT Rev | GTGCAGTAGTACTTAGTGGTC |  |  | 58.2 |
|  |  |  |  |  |
