# Supplementary figures and images for: The developmental and genetic bases of apetaly in Bocconia frutescens (Chelidonieae: Papaveraceae)
Source: EvoDevo. 2016 Aug 2;7:16. doi: 10.1186/s13227-016-0054-6 (PMC4971710; doi:10.1186/s13227-016-0054-6)

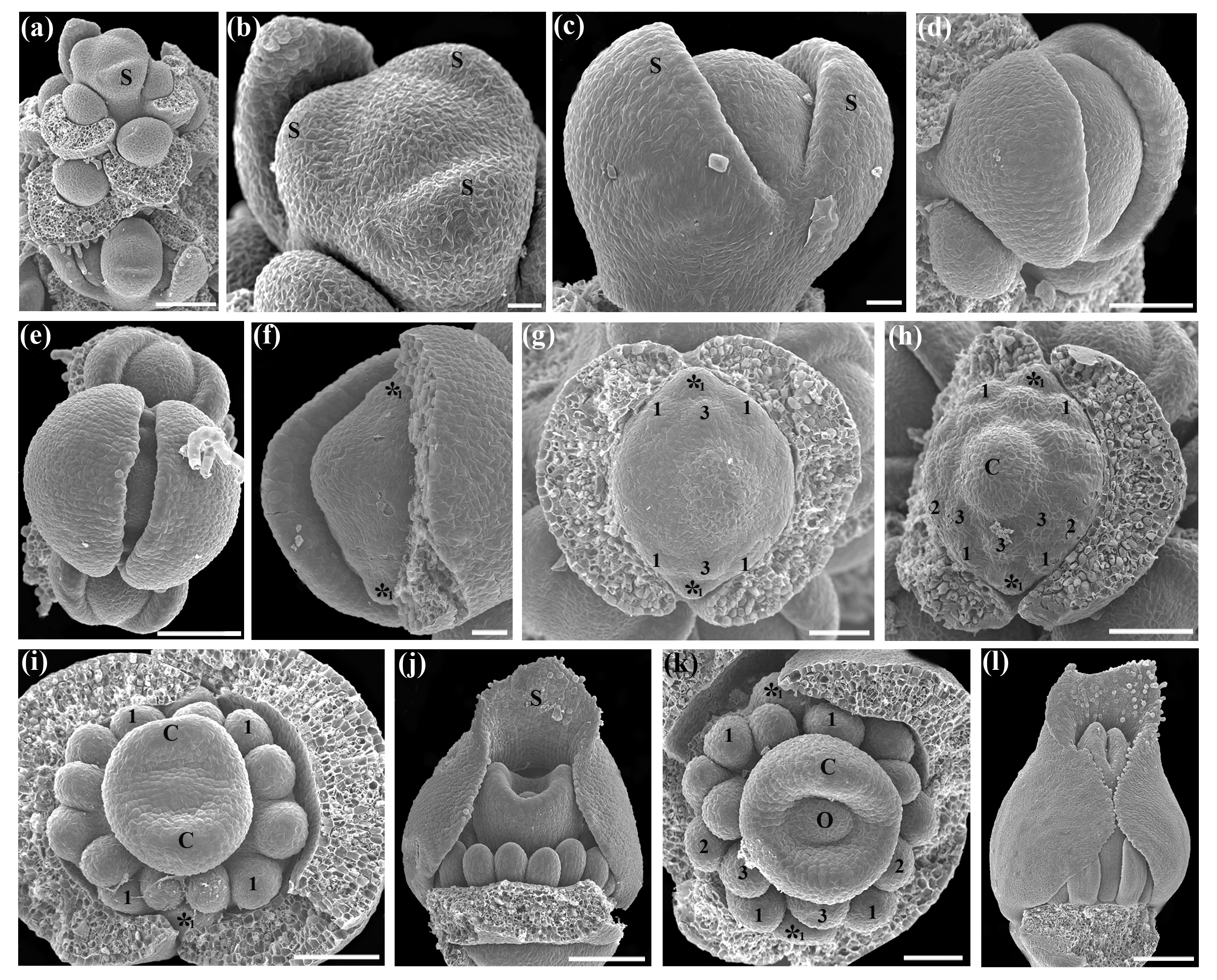

Supplement: Supplementary file 3 — 10.1186/s13227-016-0054-6. SEM micrographs of Bocconia frutescens terminal flower. A. Young inflorescence; note the terminal flower larger than the lateral flowers. B. Terminal flower with three sepals. C-E. Initiation of sepals. F. Initiation of the first whorl of homeotic stamens (*1). G. Initiation of the first whorl of true stamens alternate to the sepals (1). H-I. Initiation of the second whorl of true stamens (2) belonging to the first whorl, sometimes developed from a common primordium, followed by the third set of true stamens belonging to the second whorl (3). J-K. Initiation of the ovule. L. Late development showing a persistent sepal covering the rest of the floral organs. Bars: A, J, L = 100 µm; B, C, F = 10 µm; D, E, G-I, K = 50 µm. [file 13227_2016_54_MOESM3_ESM.tif]
